# *de novo* assembly and population genomic survey of natural yeast isolates with the Oxford Nanopore MinION sequencer

**DOI:** 10.1093/gigascience/giw018

**Published:** 2017-01-07

**Authors:** Benjamin Istace, Anne Friedrich, Léo d'Agata, Sébastien Faye, Emilie Payen, Odette Beluche, Claudia Caradec, Sabrina Davidas, Corinne Cruaud, Gianni Liti, Arnaud Lemainque, Stefan Engelen, Patrick Wincker, Joseph Schacherer, Jean-Marc Aury

**Affiliations:** 1 Commissariat à l'Energie Atomique et aux Energies Alternatives (CEA), Institut de Génomique (IG), Genoscope, BP5706, 91057 Evry, France; 2 Université de Strasbourg, CNRS, GMGM UMR 7156, F-67000 Strasbourg, France; 3 Institute of Research on Cancer and Ageing of Nice (IRCAN), CNRS UMR 7284-INSERM U1081, Faculté de Médecine, Université de Nice Sophia Antipolis, Nice, France; 4 Université d'Evry Val d'Essonne, UMR 8030, CP5706, 91057 Evry, France; 5 Centre National de Recherche Scientifique (CNRS), UMR 8030, CP5706, 91057 Evry, France

**Keywords:** *de novo* assembly, Nanopore sequencing, Oxford Nanopore, MinION device, Genome finishing, Structural variations, Transposable elements

## Abstract

Background: Oxford Nanopore Technologies Ltd (Oxford, UK) have recently commercialized MinION, a small single-molecule nanopore sequencer, that offers the possibility of sequencing long DNA fragments from small genomes in a matter of seconds. The Oxford Nanopore technology is truly disruptive; it has the potential to revolutionize genomic applications due to its portability, low cost, and ease of use compared with existing long reads sequencing technologies. The MinION sequencer enables the rapid sequencing of small eukaryotic genomes, such as the yeast genome. Combined with existing assembler algorithms, near complete genome assemblies can be generated and comprehensive population genomic analyses can be performed. Results: Here, we resequenced the genome of the *Saccharomyces cerevisiae* S288C strain to evaluate the performance of nanopore-only assemblers. Then we *de novo* sequenced and assembled the genomes of 21 isolates representative of the *S. cerevisiae* genetic diversity using the MinION platform. The contiguity of our assemblies was 14 times higher than the Illumina-only assemblies and we obtained one or two long contigs for 65 % of the chromosomes. This high contiguity allowed us to accurately detect large structural variations across the 21 studied genomes. Conclusion: Because of the high completeness of the nanopore assemblies, we were able to produce a complete cartography of transposable elements insertions and inspect structural variants that are generally missed using a short-read sequencing strategy. Our analyses show that the Oxford Nanopore technology is already usable for de novo sequencing and assembly; however, non-random errors in homopolymers require polishing the consensus using an alternate sequencing technology.

## Background

Today, long-read sequencing technology offers interesting alternatives to solve genome assembly difficulties and improve the completeness of genome assemblies, mostly in repetitive regions [1] where short-read sequencing has failed. Microbial or small eukaryotic genomes could now be assembled using Oxford Nanopore [2] or Pacific Biosciences reads alone [3, 4] or in combination with short but high quality reads [5–7]. Application of the single-molecule real-time sequencing platform to large complex eukaryotic genomes demonstrated the possibility of considerably improving genome assembly quality [8, 9]. Similar improvements were also accomplished using the 10x Genomics platform, and its application to the human genome produced encouraging results [10–12] and showed the importance of obtaining long and high-quality reads.

The most used sequencing technologies are based on the synthesis of new DNA strands, including the Illumina and Pacific Biosciences technologies [13]. These sequencing technologies based on optical detection of nucleotide incorporations are often commercialized through large-sized and expensive instruments. For example, the cost of the commercially available Pacific Biosystems RS II instrument is high and the infrastructure and implementation needs make it inaccessible to large sections of the research community. This year Oxford Nanopore Technologies Ltd (ONT, Oxford, UK) commercialized MinION, a single-molecule nanopore sequencer that can be connected to a laptop through a USB interface [14, 15]. This system is portable (close to the size of a harmonica) and low cost (currently USD 1000 for the instrument). The MinION technology is based on an array of nanopores embedded on a chip that detects consecutive 6-mers of a single-strand DNA molecule by electrical sensing [16–19]. In addition to its small size and low price, this new technology has several advantages over the older technologies. Library construction involves a simplified method, no amplification step is needed, and data acquisition and analyses occur in real time [20]. Library preparation can be performed in two ways: (i) a 10-minute library preparation based on an enzymatic method for ‘1D’ sequencing (sequencing one strand of the DNA), or (ii) a library preparation based on ligation for ‘2D’ sequencing (sequencing both the template and complement strands of the DNA). In the 2D sequencing mode, the two strands of a DNA molecule are linked by a hairpin and sequenced consecutively. When the two strands of the molecule are read successfully, a consensus sequence is built to obtain a more accurate read (called 2D read). Otherwise only the template or complement strand sequence is provided (called 1D read).

Here, we sequenced the genomes of 22 *Saccharomyces cerevisiae* isolates to determine if the MinION system could be used in population genomic projects that require a deeper view of the genetic variation landscape. Even if the throughput of MinION was still heterogeneous, we were able to perform the sequencing in a reasonable time using six MinION devices (<2 days per strain). First, we resequenced the *S. cerevisiae* S288C reference genome using a nanopore long-read sequencing strategy to evaluate recent assembly methods. We generated a complete benchmark of the assembly structures, as well as the completeness of complex regions. Next, we selected 21 strains of *S. cerevisiae* that were genetically diverse, based on preliminary results of the 1002 Yeast Genomes Project a large-scale short-read resequencing project (http://1002genomes.u-strasbg.fr/). The genomes of these 21 strains were *de novo* sequenced and assembled with Nanopore long-reads to have a better insight into the variation of their genomic architecture. We obtained near complete assembly, in terms of genes, as well as transposable elements and telomeric regions. The most contiguous assembly produced a single contig per chromosome, except for chromosomes 3 and 12, the latter containing the large repeated rDNA cluster.

## Results

### MinION data evaluation

**Figure 1:**
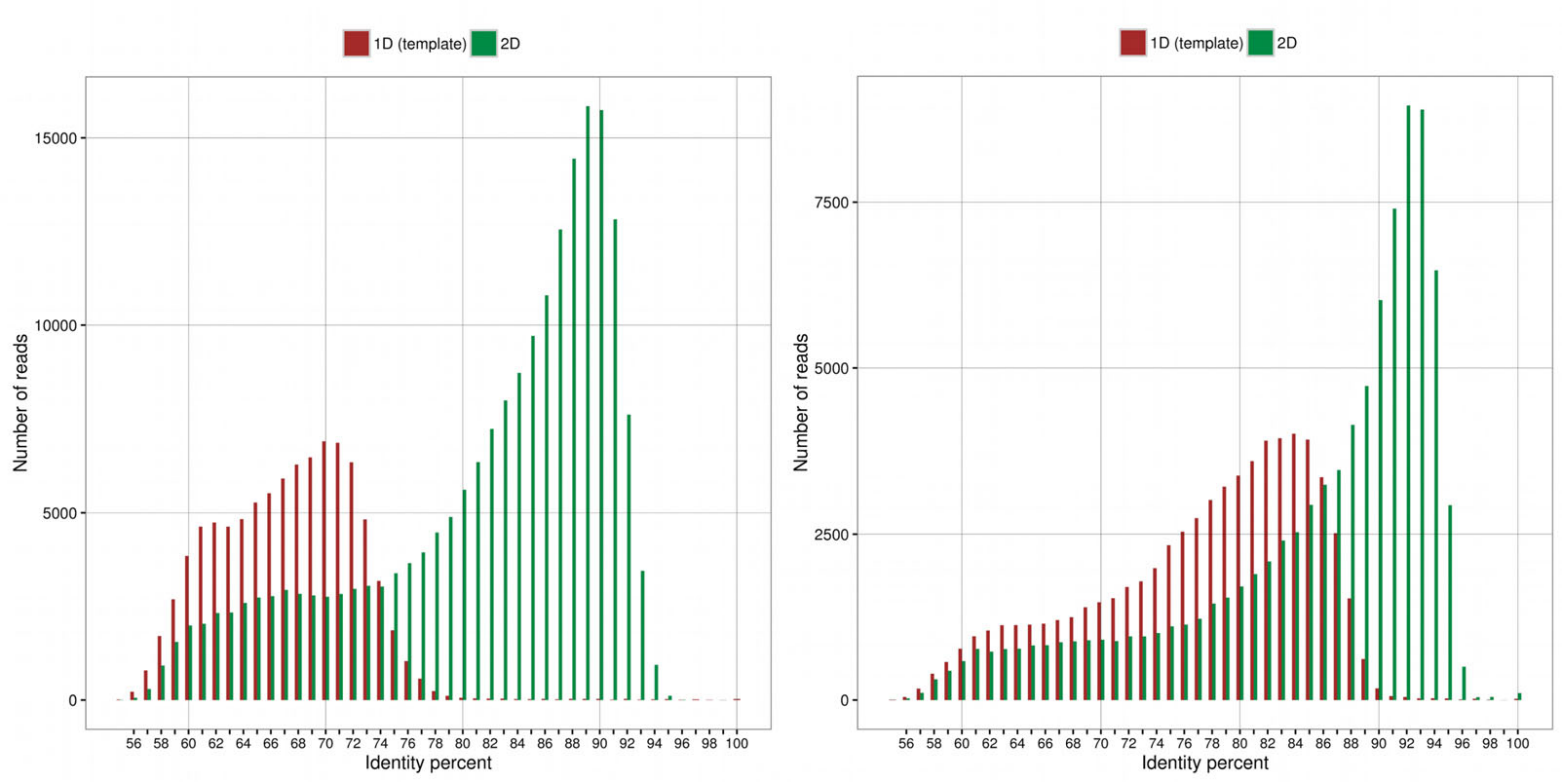
Identity distribution of Nanopore reads. Percent identity of the aligned MinION 1D (red bars) and 2D (green bars) reads. The MinION reads were aligned using LAST software. (**a**) R7.3 chemistry. (**b**) R9 chemistry.

We first sequenced the S288C genome by doing 11 MinION Mk1 runs with the R7.3 chemistry. On average, a 48-hour run produced more than 200 Mb of sequence, and the best run throughput was 400 Mb. Two 2D library types with 8 kb and 20 kb mean fragmentation sizes were used. They led to nearly 360 000 reads with a cumulative length of approximately 2.3 Gb and 63% of the nucleotides were in 2D reads, which represented a 187x and 118x genome coverage for 1D and 2D reads, respectively. Template reads had a median length of 8.9 kb, while 2D reads had a median length of 7.7 kb. All sequencing reads were aligned to the S288C reference genome using BWA [21] to assess their quality. We successfully aligned 95.6% of the 2D reads with an average error rate of 17.2% (Fig. 1a). ONT tagged high-quality 2D reads as “2D pass” reads (reads with an average per-base quality higher than 9), and 99.7% of the 2D pass reads were aligned to the reference genome with an average error rate of 12.2%. We then parsed the alignment files to search for errors in stretches of the same nucleotide (homopolymers). About 85% of A, T, C, and G homopolymers of size 2 were present correctly in the reads. This percentage decreased rapidly to 65% for homopolymers of size 4 for A and T homopolymers and to 70% for C and G homopolymers. For size 7 homopolymers, it was 30% for A and T homopolymers and 35% for C and G homopolymers (Fig. S1a).

We also sequenced the S288C genome using the R9 chemistry, the recently released version of the pore. We obtained approximately 1 Gb of reads; 568 Mb were 2D reads, which represents an 85x coverage with 1D reads and a 47x coverage with 2D reads. The mean 2D length was 6.1 kb. We aligned 82.1% of the 1D reads with a mean identity percentage of 82.8% and 94.3% of the 2D reads with a mean identity percentage of 85.2% (Fig. 1b). As we did with the R7.3 reads, we also searched for errors in homopolymers (Fig. S1b). The numbers of correct A, T, C, and G homopolymers started at about 90% for size equal to 2, then decreased to 75% for A and T homopolymers of size 4 and to 60% for the C and G homopolymers. For size 7 homopolymers, it was 32% for A, T, and C homopolymers and 35% for G homopolymers.

### Comparison of Nanopore-only assemblers

We tested Canu [22], Miniasm [23], SMARTdenovo [24], and ABruijn [25] with different subsets of 1D, 2D, and 2D pass reads (Supplementary File 2 and Table S1) and kept the most contiguous assembly for each software.

With Canu, the assembly with the higher N50 was obtained with the whole set of 2D pass reads (67x coverage). The assembly was composed of 37 contigs with a cumulative length of 12 Mb and seven chromosomes were assembled in one or two contigs. After aligning the contigs to the S288C reference genome using Quast [26], we detected a high number of deletions (120 365), which were often localized in homopolymers (58%). As a consequence, only 454 of the 6243 genes found in the assembly were insertion/deletion (indel) free (Table S2). With Miniasm, the most contiguous assembly was obtained using the 2D reads corrected by Canu, which represented coverage of approximately 108x. The Miniasm assembly was composed of 28 contigs with a cumulative length of 11.8 Mb, and 13 chromosomes were assembled in one or two contigs. The Miniasm consensus sequence contained the higher number of mismatches and indels (Table S2). With SMARTdenovo, 30x of the longest 2D reads produced the assembly with the highest contiguity. It was composed of 26 contigs, with a total length of 12 Mb, and 14 chromosomes were assembled in one or two contigs. The SMARTdenovo assembly better covered the reference genome (>99%) and contained the highest number of genes (98.8% of the 6350 S288C genes), but the Quast output again revealed a high number of deletions (128 050). With ABruijn, we obtained the assembly with the highest N50 when using all the 2D reads as input, which represented coverage of approximately 120x. The assembly contained 23 contigs with a cumulative length of 11.9 Mb, and 14 chromosomes were assembled in one or two contigs (Table S2).

Next, we aligned the assemblies (Canu, Miniasm, SMARTdenovo, and ABruijn) to the S288C reference genome using NUCmer [27], and visualized the alignments with mummerplot (Figs. S2, S3, S4, and S5). We also examined the coordinates of the alignments to search for chimera. We did not detect any chimeric contigs in the Canu, Miniasm, or SMARTdenovo assemblies; however, we did find some in the ABruijn assembly. Three chimeric contigs in the ABruijn assembly showed links between chromosomes 3 and 13 (first contig), chromosomes 3 and 2 (second contig), and chromosomes 10 and 2 (third contig). To verify that the portions of these contigs were effectively chimeric, we back aligned the Nanopore reads to the assembly and could not find any sequence that validated these links. Unsurprisingly, these three chimeric contigs were fused at Ty1 transposable element locations.

The alignment of each assembly to the reference genome showed that neither Canu, Miniasm, nor SMARTdenovo could assemble the mitochondrial (Mt) genome completely. Because ABruijn was the only assembler to assemble the complete Mt genome sequence, we decided to use it to assemble the Mt DNA of the remaining 21 yeast strains (see below).

Generally, long reads allow tandem duplicated genes to be resolve, as for instance the *CUP1* and *ENA1-2* gene families. We compared the maximum number of copies found in the Nanopore reads and the estimated number of copies based on Illumina reads coverage of these two tandem-repeated genes with the number of copies of these two genes in the four assemblies (Table S3). After aligning the paired-end reads to the reference sequence and computing of the coverage, we estimated that *CUP1* and *ENA1-2* were present in seven and four copies, respectively. The maximum numbers of copies of these genes in a single Nanopore read were eight for *CUP1* and five for *ENA1-2*. The numbers of copies of *CUP1* and *ENA1-2* were, respectively, nine and three in the Canu assembly, seven and two in the Miniasm assembly, and seven and four in the SMARTdenovo and ABruijn assemblies.

The number of indels in each assembly was considerably high for each assembler. Thus, we tested Nanopolish [2], the most commonly used Nanopore-only error corrector. We used the SMARTdenovo assembly, which was the most continuous and gene-rich assembly, and all 2D reads for this test. After the error correction step, the cumulative length of the contigs increased to 12.2 Mb and the N50 increased to 783 kb (at best it was 924 kb for the reference genome). The number of mismatches, insertions, and deletions decreased to 1930, 7707, and 17 445, respectively. The number of genes increased to 6273 complete and 2590 without an indel (Table 1).

**Table 1:** Metrics of the SMARTdenovo S288C assemblies before and after polishing with Nanopolish using R7 reads. The Nanopore 2D reads were aligned to the most continuous SMARTdenovo assembly. The alignment was given as input to Nanopolish to correct assembly errors. Metrics were obtained by aligning the pre-polishing and post-polishing version of the assembly to the reference genome using Quast.

	SMARTdenovo pre-polishing	SMARTdenovo post-polishing
# contigs	26	26
Cumulative size	12 018 244	12 204 373
N50	771 149	782 423
N90	238 808	242 444
L50	7	7
L90	16	16
# mismatches	6970	1930
# insertions	7735	7707
# deletions	128 050	17 445
# deletions in homopolymers	79 152	6869
# genes	6251 + 24 partial	6273 + 15 partial
# genes without indels	429	2590

Although all metrics were improved, the number of indels was still too high, especially in the coding regions of the genes. We decided to polish all assemblies with 2 × 250 bp Illumina paired-end reads at 300x genome coverage, using Pilon [28], to verify if the general quality of the assembly improved. The polishing step increased the N50 of each assembly, and the maximum of 816 kb was obtained with the ABruijn assembly. Pilon reduced the number of errors of each assembly, and the Canu and ABruijn assemblies had the best base quality with about 16 mismatches (15.85 and 17.88 for Canu and ABruijn, respectively) and 22 indels (22.49 and 21.76 for Canu and ABruijn, respectively) per 100 kb. The SMARTdenovo assembly contained the highest number of complete genes (6266), and the Canu assembly contained the highest number of genes without any indels (5921) (Table 2). Furthermore, we estimated the impact of the input coverage used to polish the consensus. We performed successive polishing by using subsets of Illumina reads (ranging from 25x to 300x genome coverage). We observed similar results in terms of number of mismatches and indels, regardless of the input coverage (Fig. S6).

**Table 2:** Metrics of the S288C assemblies after polishing. Assemblies were corrected using 300x of 2 × 250 bp Illumina reads as input to Pilon. The resulting corrected assembly was then aligned to the S288C reference genome using Quast.

	Spades	Canu	Miniasm	SMARTdenovo	ABruijn
Reads dataset used	Illumina PE 2 × 250 bp	2D pass	Canu-corrected	Longest 2D	2D
Coverage	300x	67x	108x	30x	120x
# reads > 10 kb	0	16 860	21 005	28 668	28 668
# contigs	376	37	28	26	23
Cumulative size	12 047 788	12 230 747	12 113 521	12 213 590	12 182 847
Genome fraction (%)	96.464	98.519	98.421	99.352	98.635
N50	149 184	610 494	736 456	783 336	816 355
N90	19 522	191 846	265 917	242 658	257 117
L50	27	8	7	7	6
L90	100	20	16	16	16
# mismatches	1126	1898	4455	4205	2138
# mismatches per 100 kb	9.47	15.85	37.23	34.27	17.88
# insertions	81	1657	3164	2384	1325
# deletions	439	1869	5208	5551	1838
# deletions in homopolymers	38	868	4248	4023	740
# indels per 100 kb	1.97	22.49	57.27	46.76	21.76
# genes	6087 + 177 partial	6241 + 32 partial	6215 + 37 partial	6266 + 33 partial	6243 + 45 partial
# genes without indels	6023	5921	5475	5881	6002

**Figure 2:**
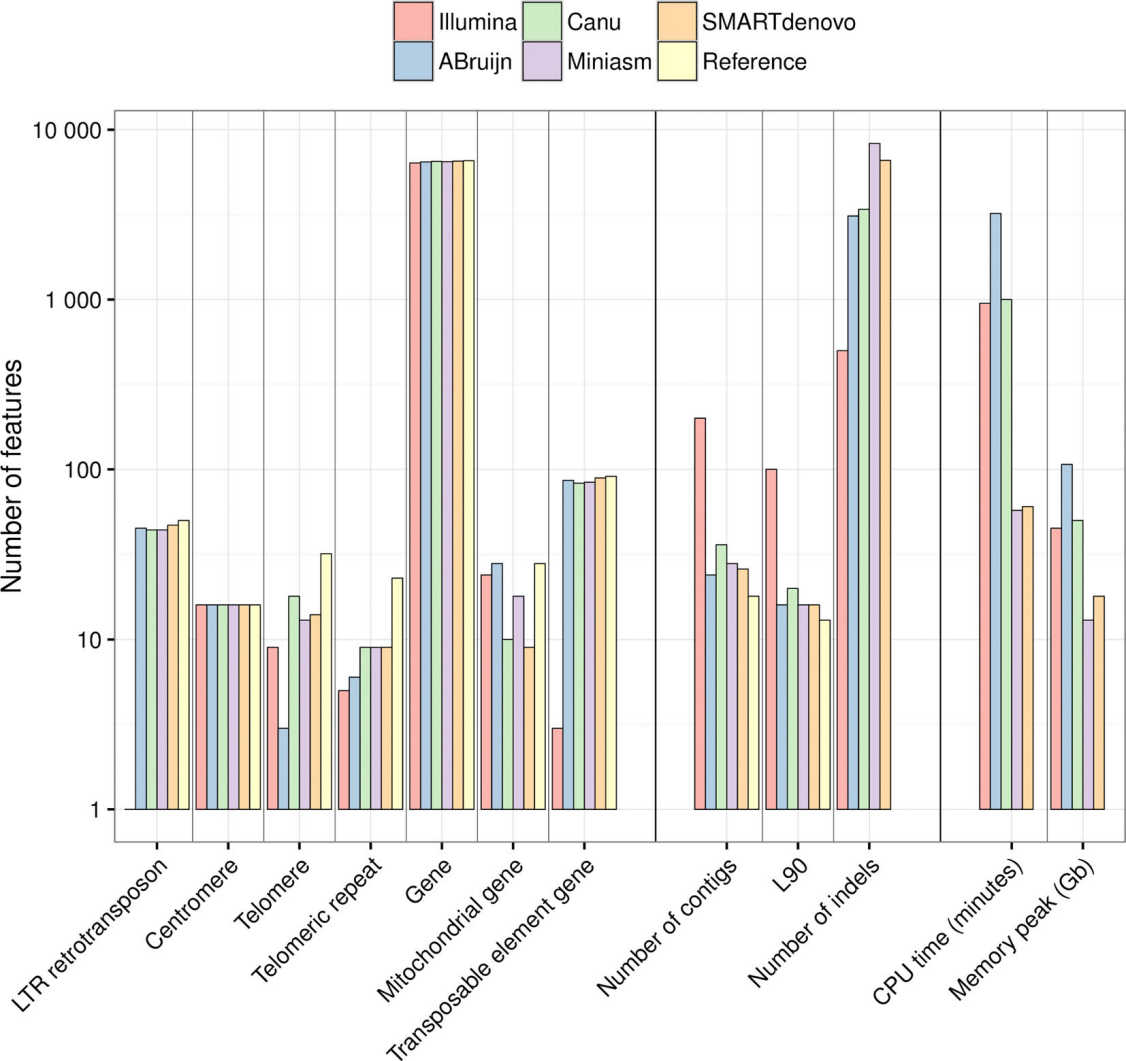
Feature composition of the S288C assemblies, assembly and quality metrics, and assembler running statistics. The feature content of the best S288C assemblies for each assembler is shown in the left part of the figure. The feature composition was obtained by aligning each assembly to the S288C reference genome. Assembly and quality metrics for each assembly, obtained by using Quast, are shown in the middle part of the figure. The running time and the memory usage of each assembler are shown in the right part of the figure.

Finally, we evaluated the composition of each assembly for various elements (genes, repeated elements, centromeres, and telomeric regions). We also generated an Illumina-only assembly using Spades assembler [29] to compare the number of features found in each assembly. All the assemblies contained nearly the same number of centromeres (120 bp regions in the reference genome assembly) and genes (Fig. 2). The Nanopore assemblies contained more complete genes than the Illumina one; however, genes without indels are more frequent in the Illumina-only assembly, although nanopore assemblies were polished using Illumina reads even between 45 and 50 Long Terminal Repeat retrotransposons (average size of 5.8 kb), while the Illumina-only assembly contained only one. The smallest number of telomeres (three) was found in the ABruijn assembly, while 9, 18, 13, and 14 telomeres were found in the Illumina, Canu, Miniasm, and SMARTdenovo assemblies, respectively. The Illumina-only assembly contained five telomeric repeats (average size 100 bp), while the Nanopore-only assemblies contained between six and nine telomeric repeats. The ABruijn assembly contained the same number of genes encoded by the mitochondrial genome as the reference sequence, because it was the only assembler to fully assemble the Mt genome.

### S288C assemblies with R9 data

The R9 version of the pore was released too late for us to use it to sequence all the natural *S. cerevisiae* isolates. However, we did produce some data to compare the R7.3 and R9 assemblies. Because SMARTdenovo produced the best results (higher continuity and higher gene content), we used it to assemble the R9 data generated from the S288C strain. We input four different read datasets: all 1D and 2D reads, only 2D reads, 30x of the longest 2D reads, or 30x of the longest 1D and 2D reads (Table S4).

This time, the 30x of the longest 1D and 2D reads dataset gave the best results. Indeed, the contiguity of the assembly increased, and the number of contigs decreased from 26 with the R7.3 assembly to 23 with the R9 assembly. The number of indels also decreased from 133 676 with the R7.3 version to 95 012 with the R9 version. A direct consequence of using the R9 version was that almost all the genes were found, and 6302 of the 6350 known genes were complete and 1226 did not contain any indels.

### Sequencing and assembly of the genomes of the 22 yeast strains

To explore the variability of the genomic architecture within *S. cerevisiae*, 21 natural isolates were sequenced in addition to the S288C reference genome using the same strategy, namely, a combination of long Nanopore and short Illumina reads. Sequenced isolates were selected to include as much diversity as possible in terms of global locations (including Europe, China, Brazil, and Japan), ecological sources (such as fermented beverages, dairy products, trees, and fruit soil), as well as genetic variation highlighted in the frame of the extensive resequencing 1002 Yeast Genomes project (http://1002genomes.u-strasbg.fr/) (Table S5). Among these isolates, the nucleotide variability was distributed across 491 076 segregating sites and the genetic diversity, estimated by the average pairwise divergence (π), was 0.0062, which is close to what is observed for the whole species [30].

A total of 78 MinION Mk1 runs were performed and the highest throughput we obtained was 650 Mb (1D and 2D reads). This led to 1.4 million of 2D reads with a cumulative length of 12 Gb. We obtained 2D coverage that ranged from 22x to 115x (Fig. S7) among the strains with a median read length of approximately 5.4 kb and a maximum size of 75 kb (Fig. S8). In general, three runs or less were sufficient to obtain the expected coverage. Next, for each strain, we gave varying coverages of the longest 2D reads (Table S6) as input to SMARTdenovo and retained the most contiguous assembly. These assemblies were then given as input to Pilon for a polishing step with around 300x of Illumina paired-end reads (each strain was individually sequenced using the Illumina technology). After polishing, we obtained a median number of contigs of 27.5 (Table 3), the minimum number was for the CEI strain (18 contigs) and the maximum was for the BAM strain (105 contigs). The median cumulative length was 11.93 Mb and ranged from 11.83 Mb for the ADQ strain to 12.2 Mb for the CNT strain. The median N50 contig size was 593 kb and varied from 201 kb for the CIC strain to 896 kb for the ADQ strain. The L90 varied from 14 for the BCN, CEI, and CNT strains to 72 for the BAM strain with a median equal to 19.5.

**Table 3:** Assembly metrics of the SMARTdenovo assemblies of all yeast strain genomes.

	# contigs	Cumul (bp)	N50 (bp)	N90 (bp)	L50	L90	Max size (bp)
ABH	22	11 960 929	803 880	267 734	6	16	1 483 918
ADM	41	11 883 044	474 542	171 488	10	26	1 009 064
ADQ	26	11 828 347	896 166	223 992	6	18	1 223 692
ADS	33	11 706 636	524 733	247 699	9	21	1 050 223
AEG	23	12 026 175	681 360	273 814	7	16	1 244 014
AKR	25	11 911 766	729 090	243 900	7	17	1 056 085
ANE	47	11 900 397	312 705	144 286	11	31	933 716
ASN	40	11 904 493	394 798	143 405	11	28	846 371
AVB	31	11 991 127	609 633	199 011	7	20	1 225 549
BAH	28	11 829 394	571 862	227 561	8	20	1 066 359
BAL	27	11 907 375	678 155	269 114	7	19	1 075 839
BAM	105	11 996 380	162 412	53 623	24	72	450 388
BCN	19	11 775 292	785 507	458 793	6	14	1 410 650
BDF	45	12 068 568	460 458	116 953	10	29	863 099
BHH	26	11 973 506	577 727	221 661	7	18	1 530 377
CBM	68	11 553 446	258 798	86 167	16	44	521 412
CEI	18	11 987 201	800 227	451 575	6	14	1 480 681
CFA	24	11 834 226	726 317	225 716	7	17	1 032 352
CFF	81	12 162 869	236 957	83 285	18	54	550 022
CIC	96	12 016 445	201 870	63 799	22	63	377 026
CNT	22	12 171 929	800 046	440 742	6	14	1 402 970
CRV (S288C)	26	12 213 584	783 337	242 658	7	16	1 532 642
Median	27.5	11 936 347	593 680	224 854	7	19.5	1 061 222
Reference	17	12 157 105	924 431	439 888	6	13	1 531 933

To assemble the mitochondrial (Mt) genome, we used all the 2D reads as input to ABruijn. As a result, we obtained an assembly for each strain and extracted the Mt genome after mapping the contigs against the reference Mt genome. As was the case for the chromosomes, we used Pilon with Illumina paired-end reads to obtain a corrected consensus sequence.

### Transposable elements

**Figure 3:**
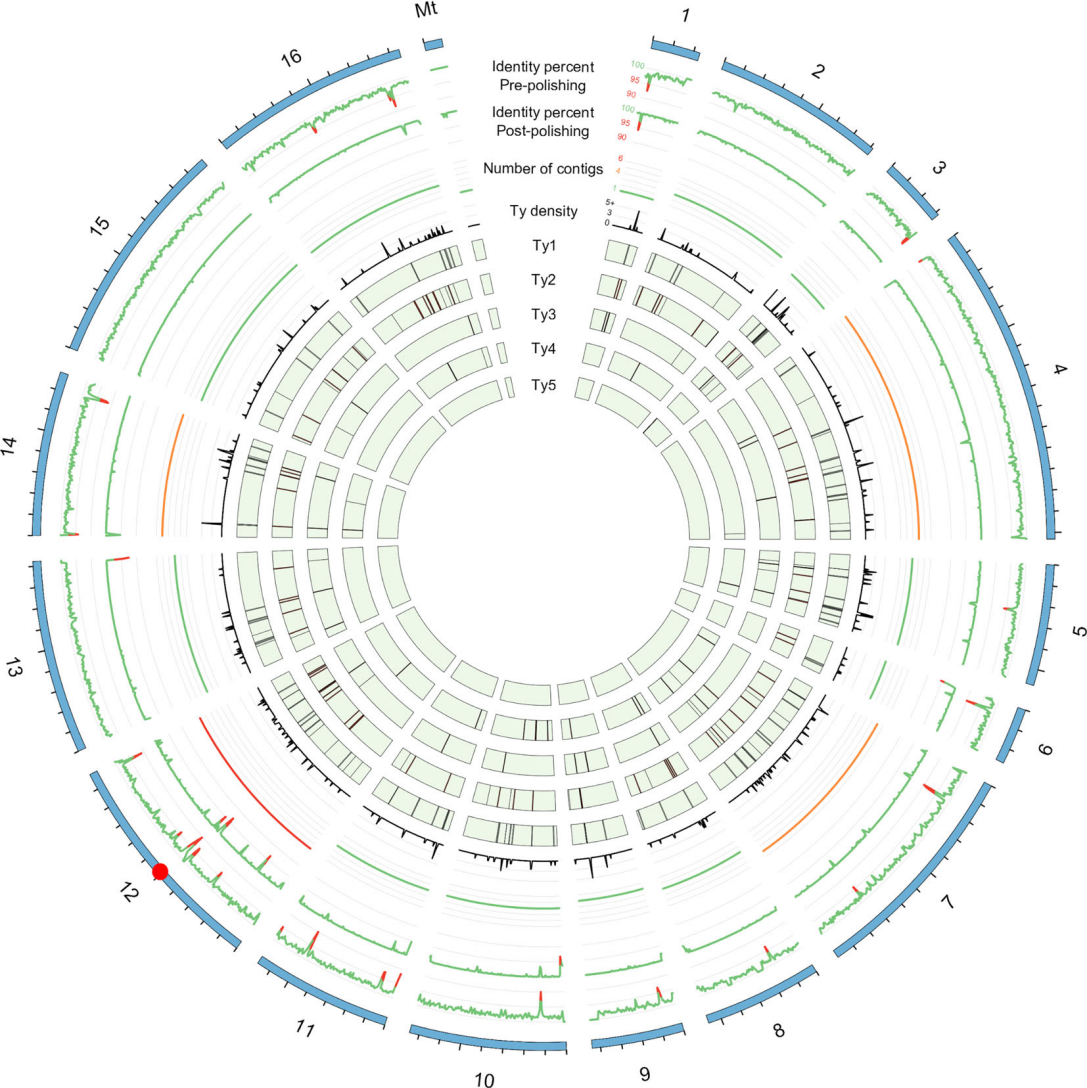
Cartography of the Ty transposon family. First and second tracks show, respectively, the percentage identity of the SMARTdenovo S288C assembly before and after polishing with Illumina paired-end reads using Pilon. The third track shows the 80th percentile number of contigs obtained for each strain and for all chromosomes. The remaining tracks show the density of Ty transposons or positions of the Ty1, Ty2, Ty3, Ty4, and Ty5 transposons across all the yeast strains. The red dot on the karyotype track shows the position of the rDNA cluster.

The availability of high-quality assemblies allowed us to establish an extensive map of the transposable elements (TEs) to obtain a global view of their content and positions within the 21 natural yeast isolates (Fig. 3). Using a reference sequence for each of the five known TE families in yeast (namely Ty1 to Ty5), we mapped the TEs in each assembled genome. Among the 50 annotated TEs in the S288C reference genome, 47 were detected at the correct chromosomal locations in our assembly, but three Ty1 locations were not recovered. Seven additional Ty1 elements were found at unannotated sites, three of them have already been detected in the reference genome [31]. These results attest to the high accuracy of our assembly strategy for TE detection and localization. Among the 22 isolates, the TE content was highly variable (Table 4), ranging from 5 to 55 elements, with a median value of 15. While the frequency of the Ty4 and Ty5 elements was clearly low in all the isolates (up to four and two elements, respectively), the Ty1, Ty2, and Ty3 elements were found in most of the isolates. The most abundant TEs were Ty1 and Ty2, except in the Chinese BAM isolate, in which 12 Ty3 elements were detected. As already described [32], the pattern of insertion of these mobile elements is either specific to a given isolate or shared by only a small number of isolates (mostly two or three). However, four insertion hotspots have been highlighted (shared by seven or more isolates) on chromosomes 2, 3, and 9. The shared insertion hotspots were generally not specific to a specific Ty family, except for the hotspot located on a subtelomeric region of the chromosome 3, which was specific to Ty5.

**Table 4:** Number of copies of multiple transposons across all yeast strains assemblies.

	Ty1	Ty2	Ty3	Ty4	Ty5
ABH	4	7	6	3	2
ADM	5	8	1	1	0
ADQ	4	7	1	2	0
ADS	1	9	0	0	1
AEG	15	7	2	1	2
AKR	4	4	4	1	1
ANE	1	5	3	2	0
ASN	13	6	0	0	0
AVB	0	29	0	0	2
BAH	0	6	1	3	0
BAL	8	0	12	0	0
BAM	4	13	6	2	1
BCN	6	0	0	0	0
BDF	13	3	3	3	1
BHH	20	12	5	4	0
CBM	3	1	0	1	0
CEI	2	20	1	0	0
CFA	8	1	1	0	1
CFF	6	6	2	0	1
CIC	6	3	1	1	0
CNT	17	6	1	1	1
CRV (S288C)	36	13	2	3	1
Reference	31	13	2	3	1

### Structural variations

Structural variations (SVs) such as copy number variants, large insertions and deletions, duplications, inversions, and translocations are of great importance at the phenotypic variation level [33]. Compared with single nucleotide polymorphisms and small indels, these variants are usually more difficult to identify, in particular because resequencing strategies have until recently focused mainly on the generation of short reads and reference-based genome analysis. Nanopore long-reads sequencing data allow the copy numbers of tandem genes to be determined. As a testbed, we focused on two loci that are known to contain multi-copy genes, namely *ENA* and *CUP1. ENA* genes encode plasma membrane Na^+^-ATPase exporters, which play a role in the detoxification of Na+ ions in *S. cerevisiae. CUP1* genes encode metallothioneins, which bind copper and are involved in resistance to copper exposure by amplification of this locus. To determine the degree of divergence among the 21 strains, we searched for the numbers of copies of the *CUP1* and *ENA*, two tandem-repeated genes in the assemblies (Table 5). For this purpose, we extracted the corresponding sequence from the S288C reference genome and aligned it to the assemblies of each strain. As expected and already reported [34], the copy numbers of *ENA1* and *CUP1* varied greatly across the strains. We found that the copy numbers of *ENA* genes in the 21 isolates ranged from 1 in 12 of the genomes to 5 in the BHH strain (Table 5). The copy numbers of *CUP1* genes fluctuated even more, ranging from 1 to 10 copies in the ABH and AEG strains. We also determined the fitness of the 21 isolates in the presence of CuSO_4_ and observed a correlation between the number of *CUP* genes and the resistance of the strain to high concentration of CuSO_4_ (Fig. S9).

**Table 5:** Copy number of *ENA1-2* and *CUP1* tandem-repeated genes across the 21 natural isolates assemblies.

	*ENA1-2*	*CUP1*
ABH	1	10
ADM	2	1
ADQ	1	1
ADS	2	3
AEG	2	10
AKR	1	1
ANE	1	1
ASN	1	3
AVB	4	2
BAH	1	1
BAL	1	1
BAM	1	2
BCN	1	1
BDF	4	4
BHH	5	3
CBM	1	1
CEI	1	1
CFA	1	1
CFF	2	4
CIC	2	4
CNT	2	1

Besides copy number variants, we also focused on larger structural variants, such as translocations and inversions, because our highly contiguous assemblies allowed us to investigate these events. We aligned the polished assemblies of the 21 strains to the reference genome using NUCmer and inspected the alignments with the mummer software suite to search for structural variations. We detected 29 translocations and 4 inversions within the assemblies of 17 strains (Table 6). The median length of an inversion was 94 kb and their breakpoints were located mostly in intergenic regions. It is well recognized that SVs might play a major role in the genetic and phenotypic diversity in yeast [35, 36]. However, up to now, it was impossible to assemble and have an exhaustive view of the SVs content in any *S. cerevisiae* natural isolates. Indeed, short-read sequencing approaches are not suitable for SVs studies, because they result in a high number of false positive as well as false negative detected events.

**Table 6:** Chromosomic rearrangements detected across all 21 strains.

Strain	Chromosome 1	Chromosome 2	Type
ABH	5	14	Translocation
ABH	5	14	Translocation
ABH	5	14	Translocation
ABH	14	14	Inversion
ADM	2	4	Translocation
ADM	5	7	Translocation
AKR	15	4	Translocation
ANE	16	5	Translocation
ANE	9	14	Translocation
ASN	5	2	Translocation
AVB	12	7	Translocation
AVB	7	12	Translocation
BAH	4	7	Translocation
BAH	10	9	Translocation
BAL	8	9	Translocation
BAM	4	7	Translocation
BAM	12	13	Translocation
BCN	6	13	Translocation
BCN	6	15	Translocation
BDF	4	14	Translocation
BDF	4	4	Inversion
BDF	5	12	Translocation
BDF	10	5	Translocation
BHH	12	12	Inversion
BHH	12	12	Inversion
CBM	16	3	Translocation
CBM	4	7	Translocation
CBM	12	15	Translocation
CEI	11	12	Translocation
CFF	14	12	Translocation
CIC	11	8	Translocation
CIC	4	7	Translocation
CNT	6	14	Translocation

Among the detected events, one translocation detected between chromosomes 5 and 14 in the ABH isolate and another translocation between chromosomes 7 and 12 in the AVB isolate have already been described and confirmed in a reproductive isolation study in *S. cerevisiae* [35]. A deeper investigation of our assemblies highlighted the presence of full-length Ty transposons at some junctions of the translocation events. For example, the complex Ty-rich junctions of the translocation between the chromosomes 7 and 12 in the ABH isolate were in complete accordance with previously reported results [35]. Our results underline the high resolution of the constructed assemblies and show that complex events, such as translocations, can be detected accurately with our strategy. Among the 22 isolates, 6 were devoid of translocation events, whereas the other 16 carry 1 to 4 such structural rearrangements compared to the reference.

However, several limitations can be highlighted for these detections. Contrary to expectations, no translocation that specifically affected subtelomeric regions was identified, underlining the difficulty of discriminating regions that are variable and contain a large number of repeated segments. Moreover, the detection accuracy is highly dependent on the completeness of the assembly, because, if translocation breakpoints are located on contigs boundaries, they will not be detectable.

### Mitochondrial genome variation

The ABruijn assembler allowed the construction of a single contig corresponding to the Mt genome for each isolate. To assess the quality of the assemblies, we aligned the polished S288C Mt contig to the reference sequence (GenBank: KP263414). Only four single nucleotide polymorphisms and few indels, representing 15 bp of cumulative length, were detected. For all but two natural isolates, all the Mt genes (8 protein coding genes, 2 rRNA subunits, and 24 tRNAs) were conserved and syntenous. The Mt genomes of the two remaining isolates (CNT and CFF) contained one and two repeated regions covering a total of 6.5 and 8 kb, respectively. In the CNT, the repeated region was in the *COX1* gene and affected its coding sequence. In the CFF isolate, the *COX1, ATP6*, and *ATP8* genes would have been tandemly duplicated. However, because we could not identify reads that clearly covered the repeated regions and then confirmed the structural variations, we excluded these two Mt genome assemblies from our dataset.

The sizes of the 20 considered assemblies ranged from 73.5 to 86.9 kb, which is close to the size reported previously [37]. The differences in size between the assemblies can mainly be attributed to the intron content of the *COX1* and *COB* genes (from two to eight introns in *COX1* and from two to six introns in *COB*). These variations lead to extensive gene length variability ranging from 5.7 kb to 14.9 kb for *COX1* and from 3.2 kb to 8.6 kb for *COB*, while the coding sequences of these two genes were exactly the same length among the 20 isolates. Intergenic regions also accumulate many small indels, including those that affect the interspersed GC-clusters and a few large indels that sometimes correspond to variable hypothetical open reading frames (ORFs), leading to sizes that range from 51.6 to 58 kb. To a lesser extent, the 21S rRNA gene is also subjected to size variation that ranges from 3.2 to 4.4 kb.

## Discussion

One of the major advantages of the Oxford Nanopore technology is the possibility of sequencing very long DNA fragments. In our analyses, we obtained 2D reads up to 75 kb in length, indicating that the system was able to read without interruption a flow of at least 150 000 nucleotides. Furthermore, the results of this analysis indicate that the error rate of the ONT R7.3 reads was in the range that is obtained using existing long-read technologies (i.e., about 15% for 2D reads). However, the errors are not random and they significantly impact stretches of the same nucleotides (homopolymers), which seems to be a feature inherent to the ONT sequencing technology. Because the pore detects six nucleotides at a time, segmentation of events is problematic in genomic regions with homopolymers longer than six bases [38]. With the current R7.3 release, homopolymers are prone to base deletion (representing 66% of the errors observed in homopolymers). It may be improved with a steadier passing speed through the pore or by increasing the speed of the molecule through the pore. In the same way, the basecaller algorithm could be optimized to increase the accuracy per base. ONT have recently reported several changes, including a fast mode (250 bp/second instead of 70 bp/second with R7.3 chemistry) and new basecaller software based on neural networks. These new features are incorporated in the R9 version of MinION. We performed R9 experiments and observed a significant decrease in the error rate (with 1D and 2D reads, Fig. 1). Using this new release, homopolymers were more prone to base insertions (representing 63% of the errors observed in homopolymers). Systematic errors are problematic for genome assembly, because they lead to the construction of less accurate consensus sequences. Furthermore, indels negatively impact gene prediction, because they can create frameshifts in the coding regions of genes. We concluded that nanopore-only assemblies are difficult to use for analysis at the gene level unless they are polished. However, polishing based only on nanopore reads was not sufficient, because although it reduced the number of indels by more than seven times, we still had about 3700 genes that were affected by potential frameshifts. The recently developed R9 chemistry greatly improved the overall quality of the consensus sequences, because starting with only 45x of 2D reads we obtained an assembly with the same contiguity but with a decrease of nearly 30% in the number of indels (95 012 compared with 133 676). We consider that the ONT sequencing platform will evolve in the coming years to produce high-quality long reads. Until then, a mixed strategy using high-quality short reads remains the only way to obtain high-quality consensus sequences as well as a high level of contiguity. Indeed, for the assembly of repetitive regions, the nanopore-only assemblies outperformed the short-reads assemblies.

Our benchmark of nanopore-only assemblers shows that, unfortunately, a single “best assembler” does not exist. Canu reconstructed the telomeric regions better and provided a consensus of higher quality than Miniasm and SMARTdenovo. ABruijn seemed to produce the most continuous assembly but some of the contigs were chimeric. However, ABruijn was the only assembler to fully assemble the mitochondrial genome, and that is why we chose it to assemble the Mt genomes of the 22 yeast strains. SMARTdenovo provided good overall results for repetitive regions, completeness, contiguity, and speed. It was the most appropriate choice to assemble the genome of all the yeast strains even if its major drawback was the absence of the Mt genome sequence among the contig output.

The high contiguity of the 22 nanopore-only assemblies allowed us to detect transposable element insertions and to provide a complete cartography of these elements. Ty1 was the most abundant element and it was spread across the entire genome. Chromosome 12 was always the most fragmented in our assemblies due to the presence of the rDNA cluster (around 100 copies in tandem). Furthermore, we easily identified known translocations (between chromosomes 5 and 14 in the ABH isolate and between chromosomes 7 and 12 in the AVB isolate). The high contiguity of the assemblies seemed to be limited by the read size rather than the error rate. Work is still needed to prepare high-weight molecular DNA, enriched in long fragments. The yeast genomes were successfully assembled with 8-kb and 20-kb fragment-sized libraries, but more complex genomes will require longer reads.

### Methods

#### DNA extraction

Yeast cells were grown on YPD media (1% yeast extract, 2% peptone, and 2% glucose) using liquid culture or solid plates. Total genomic DNA was purified from 30 ml YPD culture using Qiagen Genomic-Tips 100/G and Genomic DNA Buffers as per the manufacturer's instructions. The quantity and quality of the extracted DNA were controlled by migration on agarose gel, spectrophotometry (NanoDrop ND-1000, ThermoFisher, Wilmington, DE, USA), and fluorometric quantification (Qubit, ThermoFisher, Wilmington, DE, USA).

### Illumina PCR-free library preparation and sequencing

DNA (6 μg) was sonicated to a 100- to 1500-bp size range using a Covaris E210 sonicator (Covaris, Woburn, MA, USA). Fragments were end-repaired using the NEBNext End Repair Module (New England Biolabs, Ipswich, MA, USA) and 3΄-adenylated with the NEBNext dA-Tailing Module. Illumina adapters were added using the NEBNext Quick Ligation Module. Ligation products were purified with AMPure XP beads (Beckmann Coulter Genomics, Danvers, MA, USA). Libraries were quantified by qPCR using the KAPA Library Quantification Kit for Illumina Libraries (KapaBiosystems, Wilmington, MA, USA), and library profiles were assessed using a DNA High Sensitivity LabChip kit on an Agilent Bioanalyzer (Agilent Technologies, Santa Clara, CA, USA). Libraries were sequenced on an Illumina MiSeq or a HiSeq 2500 instrument (San Diego, CA, USA) using 300 or 250 base-length read chemistry in a paired-end mode.

### Nanopore 20-kb libraries preparation

MinION sequencing libraries were prepared according to the SQK-MAP005 or SQK-MAP006-MinION gDNA Sequencing Kit protocols. Six to 10 μg of genomic DNA was sheared to approximately 20 000 bp with g-TUBE (Covaris, Woburn, MA, USA). After clean-up using 0.4x AMPure XP beads, sequencing libraries were prepared according to the SQK-MAP005 or SQK-MAP006 Sequencing Kit protocols, including the PreCR treatment (NEB, Ipswich, MA, USA) for the SQK-MAP005 protocol or the NEBNext FFPE DNA repair step (NEB) for the SQK-MAP006 protocol.

### Nanopore 8-kb libraries preparation

MinION sequencing libraries were prepared according to the SQK-MAP005 or SQK-MAP006-MinION gDNA Sequencing Kit protocols. Two μg of genomic DNA was sheared to approximately 8000 bp with g-TUBE. After clean-up using 1x AMPure XP beads, sequencing libraries were prepared according to the SQK-MAP005 or SQK-MAP006 Sequencing Kit protocol, including the PreCR treatment for the SQK-MAP005 protocol or the NEBNext FFPE DNA repair step for the SQK-MAP006 protocol.

### Nanopore low-input 8-kb libraries preparation

The following protocol was applied to some samples (Supplementary File 3). Five hundred ng of genomic DNA was sheared to approximately 8000 bp with g-TUBE. After clean-up using 1x AMPure XP beads and the NEBNext FFPE DNA repair step, 100 ng of DNA was prepared according to the Low Input Expansion Pack Protocol for genomic DNA.

### MinION flow cell preparation and sample loading

The sequencing mix was prepared with 8 μl of the DNA library, water, the fuel mix, and the running buffer according to the SQK-MAP005 or the SQK-MAP006 protocols. The sequencing mix was added to the R7.3 flowcell for a 48-hour run. The flowcell was then reloaded three times according to the following schedule: 5 hours (4 μL of DNA library), 24 hours (8 μL of DNA library), and 29 hours (4 μl of DNA library). Regarding the low-input libraries, the flowcell was loaded and then reloaded after 24 hours of run time with a sequencing mix containing 10 μl of the DNA library (Supplementary File 3).

### MinION sequencing and reads filtering

Read event data generated by MinKNOW control software (version 0.50.1.15 to 0.51.1.62) were base-called using the Metrichor software (version 2.26.1 to 2.38.3). The data generated (pores metrics, sequencing, and base-calling data) by MinION software were stored and organized using a Hierarchical Data Format. Three types of reads were obtained: template, complement, and two-directions (2D). The template and complement reads correspond to sequencing of the two DNA strands. Metrichor combines template and complement reads to produce a consensus (2D) sequence [39]. FASTA reads were extracted from MinION Hierarchical Data Format files using poretools [40]. To assess the quality of the MinION reads, we aligned reads against the *S. cerevisiae* S288C reference genome using the LAST aligner (version 588) [41]. Because the MinION reads are long and have a high error rate, we used a gap open penalty of 1 and a gap extension penalty of 1.

### Illumina reads processing and quality filtering

After the Illumina sequencing, an in-house quality control process was applied to the reads that passed the Illumina quality filters. The first step discards low-quality nucleotides (Q < 20) from both ends of the reads. Next, Illumina sequencing adapters and primer sequences were removed from the reads. Then, reads shorter than 30 nucleotides after trimming were discarded. These trimming and removal steps were achieved using in-house-designed software based on the FastX package [42]. The last step identifies and discards read pairs that mapped to the phage phiX genome, using SOAP [43] and the phiX reference sequence (GenBank: NC_001422.1). This processing resulted in high-quality data and improvement of the subsequent analyses.

### Assembler evaluation

To determine the assembler to use on the *de novo* sequenced 22 yeast strains, tests were conducted on S288C, the only *S. cerevisiae* strain for which there is an established reference genome. We used different subsets of the reads as input to Canu (github commit ae9eecc), Miniasm (github commit 17d5bd1), SMARTdenovo (github commit 61cf13d), and ABruijn (github commit dc209ee), four assemblers that can take advantage of long reads. These subsets consisted of varying coverages of 1D, 2D, 2D pass reads, which are 2D reads that have an average quality greater than nine, and reads corrected by Canu. Canu was executed with the following parameters: genomeSize = 12 m, minReadLength = 5000, mhapSensitivity = high, corMhapSensitivity = high, errorRate = 0.01, and corOutCoverage = 500. Miniasm was run with the default parameters indicated on the github web site. SMARTdenovo was executed with the default parameters and –c 1 to run the consensus step. ABruijn was run with default parameters. After the assembly step, we polished each set of contigs with Pilon (version 1.1.12) using 300x of Illumina 2 × 250 bp paired-end reads. Assemblies were aligned to the S288C reference genome using Quast in conjunction with the GFF file of S288C to detect assembly errors and complete and partial genes. We also visualized the alignments using mummerplot to detect chimeric contigs.

### Assembly of the genome of the 22 yeast strains

The 22 genomes were assembled by utilizing varying sequencing coverage, going from 10x to 50x, of the longest 2D reads as input to SMARTdenovo with the default parameters and –c 1 to run the consensus step. Then, for each strain, the most contiguous assembly (based on the N50 and the number of contigs) was polished using ∼300x of 2 × 250 bp Illumina paired-end reads (each yeast strain was sequenced separately beforehand).

### Genes and transposons detection

To detect genes and transposons in the assemblies, we extracted the corresponding sequences from the reference genome. We then mapped these elements to the assemblies using the Last aligner. Only alignments that showed more than 80% identity over at least 90% of the sequence length were retained and considered as a match. We used a similar procedure to count the maximum number of genes in the Nanopore reads dataset; the only modification was that the percentage identity had to be at least 70% to account for the high error rate of the reads. To estimate the number of copies in the Illumina reads, we aligned paired-end reads to the reference genome with BWA aln and then computed the coverage using samtools mpileup algorithm [44] and divided the number we obtained for each region of interest by the median coverage of the corresponding chromosome.

### Feature number estimation

We generated an Illumina-only assembly using Spades version v3.7.0 with default parameters and compared the completeness of this assembly to the nanopore-only assemblies. To estimate the number of features across all S288C assemblies, we aligned each post-polishing consensus sequence to the S288C reference genome using NUCmer. Only the best alignments were conserved by using the *delta-filter -1* command. Next, we used the bedtools suite [45] with the command *bedtools intersect -u -wa -f 0.99* to compare the alignments to the reference GFF file. Finally, we counted the number of features of our interest.

### Circularization of mitochondrial genomes

To circularize the Mt genomes, we split the contig corresponding to the Mt sequence in each strain into two distinct contigs. Then, we gave the two contigs as input to the minimus2 [46] tool from the AMOS package. As a result, we obtained a single contig that did not contain the overlap corresponding to the circularization zone. Finally, to start the Mt sequence of all isolates at the same position as the reference, we mapped each Mt sequence to the reference using NUCmer. The *show-coords* command allowed us to identify the position in the Mt sequences of all the strains that corresponded to the first position of the reference Mt genome.

## Declarations

### Availability of data and materials

The 22 genome assemblies are freely available at http://www.genoscope.cns.fr/yeast. The Illumina and MinION data are available in the European Nucleotide Archive under accession number ERP016443. Supporting data are also available from the *GigaScience* GigaDB repository [47].

### Additional files

All the supporting data are included as three additional files: the first one contains Figs. S1–S9 and Tables S1–S6 and two excel files contain the metrics of all assemblies generated in this study and the description of each MinION run.

**Additional file 1: Figure S1.** Percentage of correct A, T, C, and G homopolymers in Nanopore 2D reads in either R7 reads (a) or R9 reads (b).

**Additional file 2: Figure S2.** Alignment of the Canu assembly. We aligned the most continuous Canu assembly to the reference genome using nucmer and visualized the alignment using the mummer software suite.

**Additional file 3: Figure S3.** Alignment of the Miniasm assembly. We aligned the most continuous Miniasm assembly to the reference genome using nucmer and visualized the alignment using the mummer software suite.

**Additional file 4: Figure S4.** Alignment of the SMARTdenovo assembly. We aligned the most continuous SMARTdenovo assembly to the reference genome using nucmer and visualized the alignment using the mummer software suite.

**Additional file 5: Figure S5.** Alignment of the ABruijn assembly. We aligned the most continuous ABruijn assembly to the reference genome using nucmer and visualized the alignment using the mummer software suite.

**Additional file 6: Figure S6.** Impact of the input coverage used to polish the nanopore-only consensus.

**Additional file 7: Figure S7.** Nanopore 2D reads coverage distribution across all yeast strains. In total 95 MinION MkI runs were done. We obtained a 2D read coverage fluctuating between 25x and 120x.

**Additional file 8: Figure S8.** Reads length distribution of 2D reads across all yeast strains.

**Additional file 9: Figure S9.** Fitness of the 21 yeast isolates in the presence of CuSO_4_ as a function of the detected number of *CUP* genes in each strain.

**Additional file 10: Table S1.** Metrics of the reads sets that lead to the best S288C assembly for each software.

**Additional file 11: Table S2.** Metrics of S288C assemblies. Varying coverages of 2D reads and reads corrected by Canu were given as input to Canu, Miniasm, SMARTdenovo, and ABruijn. Only the most contiguous assembly is shown below. Metrics were obtained by aligning the assemblies to the reference genome using Quast.

**Additional file 12: Table S3.** Number of copy of *CUP1* and *ENA1-2* tandem-repeated genes. Second column indicates the expected number of copy based on the alignment of Illumina reads on the reference. Other columns indicate either the maximum number found in Nanopore reads or in assemblies.

**Additional file 13: Table S4.** Comparison of SMARTdenovo assemblies using R9 reads.

**Additional file 14: Table S5.** Description of the studied isolates.

**Additional file 15: Table S6.** Metrics of the reads sets that lead to the best SMARTdenovo assembly for each strain. (DOCX 1008 kb).

**Additional file 16:** Supplementary_File2.xlsx. (XLXS 44 kb).

**Additional file 17:** Supplementary_File3.xlsx. (XLXS 15 kb).

### Abbreviations

LTR, long terminal repeat – MAP, MinION Access Programme – MRT, single-molecule real-time sequencing – Mt, mitochondrial – ONT, Oxford Nanopore Technology – ORF, open reading frame – USB, universal serial bus

### Ethics approval and consent to participate

Not applicable.

### Consent for publication

Not applicable.

### Competing interests

The authors declare that they have no competing interests. Oxford Nanopore Technologies Ltd contributed to this study by providing some of the R9 reagents free of charges. BI, SD, CCR, AL, SE, PW, and JMA are part of the MinION Access Programme (MAP).

### Funding

This work was supported by the Genoscope, the Commissariat à l'Energie Atomique et aux Energies Alternatives (CEA), France Génomique (ANR-10-INBS-09-08), and the Agence Nationale de la Recherche (ANR-16-CE12-0019).

### Author's contributions

CCA extracted the DNA. EP, OB, CCR, and AL optimized and performed the sequencing. BI, AF, LDA, SF, SD, SE, and JMA performed the bioinformatic analyses. BI, AF, JS, and JMA wrote the article. GL, PW, JS, and JMA supervised the study.

## Supplementary Material

GIGA-D-16-00110_Original_Submission.pdf

GIGA-D-16-00110_Revision_1.pdf

GIGA-D-16-00110_Revision_2.pdf

Response_to_Reviewer_Comments_Original_Submission.pdf

Response_to_Reviewer_Comments_Revision_1.pdf

Reviewer_1_Original_Submission_(attachment).pdf

Reviewer_1_Report_Original_Submission.pdf

Reviewer_2_Original_Submission_(attachment).pdf

Reviewer_2_Report_Original_Submission.pdf

Reviewer_2_Report_Revision_1.pdf

Reviewer_2_Revision_1_(attachment).pdf

Reviewer_3_Report_Original_Submission.pdf

Supplemental material **Additional file 1: Figure S1.** Percentage of correct A, T, C, and G homopolymers in Nanopore 2D reads in either R7 reads (a) or R9 reads (b).
**Additional file 2: Figure S2.** Alignment of the Canu assembly. We aligned the most continuous Canu assembly to the reference genome using nucmer and visualized the alignment using the mummer software suite.
**Additional file 3: Figure S3.** Alignment of the Miniasm assembly. We aligned the most continuous Miniasm assembly to the reference genome using nucmer and visualized the alignment using the mummer software suite.
**Additional file 4: Figure S4.** Alignment of the SMARTdenovo assembly. We aligned the most continuous SMARTdenovo assembly to the reference genome using nucmer and visualized the alignment using the mummer software suite.
**Additional file 5: Figure S5.** Alignment of the ABruijn assembly. We aligned the most continuous ABruijn assembly to the reference genome using nucmer and visualized the alignment using the mummer software suite.
**Additional file 6: Figure S6.** Impact of the input coverage used to polish the nanopore-only consensus.
**Additional file 7: Figure S7.** Nanopore 2D reads coverage distribution across all yeast strains. In total 95 MinION MkI runs were done. We obtained a 2D read coverage fluctuating between 25x and 120x.
**Additional file 8: Figure S8.** Reads length distribution of 2D reads across all yeast strains.
**Additional file 9: Figure S9.** Fitness of the 21 yeast isolates in the presence of CuSO_4_ as a function of the detected number of *CUP* genes in each strain.
**Additional file 10: Table S1.** Metrics of the reads sets that lead to the best S288C assembly for each software.
**Additional file 11: Table S2.** Metrics of S288C assemblies. Varying coverages of 2D reads and reads corrected by Canu were given as input to Canu, Miniasm, SMARTdenovo, and ABruijn. Only the most contiguous assembly is shown below. Metrics were obtained by aligning the assemblies to the reference genome using Quast.
**Additional file 12: Table S3.** Number of copy of *CUP1* and *ENA1-2* tandem-repeated genes. Second column indicates the expected number of copy based on the alignment of Illumina reads on the reference. Other columns indicate either the maximum number found in Nanopore reads or in assemblies.
**Additional file 13: Table S4.** Comparison of SMARTdenovo assemblies using R9 reads.
**Additional file 14: Table S5.** Description of the studied isolates.
**Additional file 15: Table S6.** Metrics of the reads sets that lead to the best SMARTdenovo assembly for each strain. (DOCX 1008 kb).
**Additional file 16:** Supplementary_File2.xlsx. (XLXS 44 kb).
**Additional file 17:** Supplementary_File3.xlsx. (XLXS 15 kb).
