# Supplementary material for: de novo assembly and population genomic survey of natural yeast isolates with the Oxford Nanopore MinION sequencer
Source: Gigascience. 2017 Jan 7;6(2):1–13. doi: 10.1093/gigascience/giw018 (PMC5466710; doi:10.1093/gigascience/giw018)
Supplement: Reviewer_1_Original_Submission_(attachment).pdf [file giw018_Reviewer_1_Original_Submission_(attachment).pdf]

Reviewer's report

Title: Developmental coordination disorder in children - experimental work and data annotation

Version:2**Date:**3 February 2015

Reviewer: Susanne Passow

Reviewer's report:

Summary

The purpose of this Data Note is to provide electrophysiological data recorded during a multisensory oddball paradigm to investigate differences between children with developmental coordination disorder (DCD) and healthy controls. The data provided comprise a set of EEG data of in total 19 subjects, grouped into three groups of relatively low sample size. In addition, information about reaction time and response accuracy as well as performance scores (motoric percentiles) in a motor test are given. In general, I think this data set could be of potential interest for GigaScience. However, I think additional data should be recorded to increase the utility of the dataset. Furthermore, I have listed additional comments that need to be addressed.

Major Compulsory Revisions

Point 1

A brief section about the theoretical background reviewing some literature in the field is missing. For instance, as a reader it would be interesting what EEG signatures/ features have already been used in previous studies to differentiate between children with and without developmental coordination disorder (DCD), at least 1-2 examples.

Point 2

It does not come across clearly whether the dataset has been analysed and published before. If yes, please provide a reference, this would be helpful for a potential user. If not, it would still be interesting to know more about the initial aim of the data collection or was the initial aim to provide the dataset for public use.

Point 3

The section about ‘Findings’ in the abstract should inform in a more detailed way about the data types provided. The ‘Conclusion’ should provide more information about potential uses and implications for the research field. Please revise.

Point 4

A dataset of N=19, per se, is not particularly large-scale in the field of EEG studies. Further, this dataset is divided into three groups, one group of children with DCD diagnosis (N=7), a group of children with suspected DCD (N=3), and a

healthy control group (N=7). In two children motor test data is missing. To detect solid group differences and condition x group interactions, which are desirable when aiming at using the ERP as a diagnostic marker, the present sample size is too small. For instance, aiming for a medium effect size of Cohen's f= 0.25 (#=0.05) and a power of 0.85 for condition x group interaction effects, one would need a total sample size of 48 subjects, thus at least 16 subjects per group. Consequently, I suggest to record additional datasets to increase the utility of the dataset.

Point 5

The definition of the groups is not clear enough in the section about 'Participants'. Please provide the information about the motoric percentiles and the cut-off values for the different groups already here. What was the motor test about? Please provide more details.

Point 6

The section about 'Data description' needs to be improved. It is a bit misleading to read something about the recording hardware under a subheading of 'Experimental Design'. Maybe you can apply the following headings and order: 1. Theoretical background and purpose of the study; 2. Participants; 3. Experimental Procedure; 4. EEG data recording. With respect to information about the EEG data recording please see Point 8.

Point 7

Could you please provide more detailed information about the hearing impairment of the participants? Were all hearing thresholds # 25 dB HL or not? If 75 dB SPL was set as an upper limit and the thresholds were higher 25 dBHL, some of the children might have gotten higher intensity increments than others. As this is partly an auditory task, this might have influenced the results. Please explain.

Was the study approved by an ethical committee? Please provide information.

Point 8

When describing the ERP data recording, you should consider the very useful guidelines provided by Picton et al. 2000 in Psychophysiology facilitating the replicability of ERP results.

Picton, T. W., Bentin, S., Berg, P., Donchin, E., Hillyard, S. A., Johnson, R., . . . Taylor, M. J. (2000). Guidelines for using human event-related potentials to study cognition: Recording standards and publication criteria. Psychophysiology, 37(2), 127-152.

For instance, I am missing information about the timing of the stimuli (trial length, response interval; inter-stimulus interval) and about breaks between the blocks. More detailed information about the kind of electrodes and the preparation of the EEG should be provided. Further information about the filtering characteristics of the recording system (i.e. use of an online bandpass filter) should be provided.

How were the eye blinks determined.

Point 9

I am not an expert on data security, but is it allowed to provide non-anonymized data as name and e-mail addresses in such a context?

Point 11

Is the percentage of eye blinks based on the number of total trials, thus 600? More informative would be if the percentage of eye blinks varied significantly across conditions and across groups. Could you provide this information in addition?

Minor Essential Revisions

- Abstract: Please do not cite references in the abstract

- Figure 1: Please add in the figure caption that the electrodes have been attached in the 10-20 system.

- Figure 2 is not carrying any important information. I don't think it is necessary.

- Figure 3: Figure caption should notify that the percentage of eye-blink artifacts is presented for each participant and not for each experiment. Also see my point 11.

Susanne Passow

Level of interest: An article of importance in its field

Quality of written English: Acceptable

Statistical review: No, the manuscript does not need to be seen by a statistician.

Declaration of competing interests:

I declare that I have no competing interests.

Susanne Passow
