# Supplementary material for: de novo assembly and population genomic survey of natural yeast isolates with the Oxford Nanopore MinION sequencer
Source: Gigascience. 2017 Jan 7;6(2):1–13. doi: 10.1093/gigascience/giw018 (PMC5466710; doi:10.1093/gigascience/giw018)
Supplement: Reviewer_2_Original_Submission_(attachment).pdf [file giw018_Reviewer_2_Original_Submission_(attachment).pdf]

Reviewer's report

Title: Developmental coordination disorder in children - experimental work and data annotation

Version:4**Date:**31 August 2016

Reviewer: Melissa Pangelinan

Reviewer's report:

I appreciate the author’s attention to the comments/changes to the manuscript in response to the reviewers. I believe that the clarity of the methods is much improved. There are a few concerns that remain.

Major Compulsory Revision:

1. Common motor skill assessments that are used in this population, like the MABC-2, BOT-2, or TGMD-2, are relatively fast to administer (~30-45 minutes). Although setting up an EEG cap with 19 electrodes may be very fast, I do not think that the speed alone justifies the use of EEG as a replacement for traditional behavioral assessments. It would be worthwhile to also add the approximate total time (set up + paradigm) and to substantiate the utility of EEG. What information is gained that cannot be gained using behavioral assessments?

Moreover, the expense of these behavioral assessments is much reduced compared with most EEG systems (i.e., ~$1000 or less compared with $80,000 for a BrainVision EEG system). As such, the important thing about the use of EEG systems is that they are able to do a better job of discriminating differences or add something unique to our understanding of DCD and the potential comorbidities. These points, the time and cost considerations, could be highlighted in the introduction or discussion.

2. Given the comorbidity of DCD and other developmental/learning disabilities (e.g., Autism Spectrum Disorder, ADHD, developmental dyslexia), it would be worthwhile to add in the metadata or participant information the existence of comorbidities and how these comorbidities were assessed (e.g., parent report, neuropsychological/educational tests, physician diagnosis). Without this information: A) it would be difficult to use this data repository to reliably combine data from different research groups and B) it would be impossible to use data-driven discrimination procedures to predict group membership (e.g., control, probably DCD, DCD, DCD+ADHD, DCD+ASD, etc.). Based on the introduction, I believe that the ultimate goal would be to address both A and B.

3. The total scores from the MABC-2 are not typically reported. The percentiles are more meaningful because the red zone is characterized as at or below the 5th percentile, the amber zone is between the 6th and 15th percentile, and the green is at or above the 15th percentile. As such, the description of the MABC under 1.2 Participants should be changed to reflect the language in the MABC-2

manual (see above or page 176 of the manual). Also, the table provides “Sum SS” which should be changed to “Total Score”.

Minor Compulsory Revisions:

1. In the table, age in months should be used instead of age in years.

2. Were the participants with visual impairments wearing corrective lenses during the task?

3. Blink artifacts are easily removed without data loss with algorithms like independent components analysis. These algorithms are now included in EEGlab or BrainVision Analyzer 2 and are very useful for young children and those that tend to blink a lot. Given a reduction in data loss, the paradigm may be able to be shortened. The authors could provide this as a suggestion.

Level of interest: An article whose findings are important to those with closely related research interests

Quality of written English: Acceptable

Statistical review: No, the manuscript does not need to be seen by a statistician.

Declaration of competing interests:

I declare that I have no competing interests.
