# Supplementary material for: de novo assembly and population genomic survey of natural yeast isolates with the Oxford Nanopore MinION sequencer
Source: Gigascience. 2017 Jan 7;6(2):1–13. doi: 10.1093/gigascience/giw018 (PMC5466710; doi:10.1093/gigascience/giw018)
Supplement: Reviewer_2_Revision_1_(attachment).pdf [file giw018_Reviewer_2_Revision_1_(attachment).pdf]

Reviewer's report

Title: Developmental coordination disorder in children - experimental work and data annotation

Version:2**Date:**12 February 2015

Reviewer: Melissa Pangelinan

Reviewer's report:

The manuscript is clear and the data collection methods are sound. There is certainly a lack of neuroimaging studies that examine children with DCD and making these data available to the public is important. However, there are several issues that need to be addressed in order for these data to be of use to other researchers (both for the metadata and in the manuscript).

Major Compulsory Revisions

1) I am not sure if traditional results are necessary in a data note submission but future users would greatly benefit from at least one data visualization to determine if these data are of sufficient quality. A figure with the individual ERPs for each group (not the group grand-average ERPs) for each condition would be particularly useful. This would provide users with an idea of the variability of these data, particularly for the children with DCD.

2) 1.1 (page 1): It is not clear why this task is appropriate for understanding the deficits affecting children with DCD. At least one statement regarding deficits in multi-sensory integration and movement time deficits in children with DCD should be included in the purpose statement.

3) 1.2.5 (page 2): Please include a table with participant details. Given the small sample size and the inherent variability of pediatric data, it would be worthwhile to include this information for ALL participants (and not just the children with DCD or suspected DCD). This table should include: age, sex, MABC raw and percentile scores for the total score and component scores (manual dexterity, ball skills, and balance scores), the degree of hearing impairment, the auditory threshold levels, and whether the child had vision correction.

Minor Essential Revisions

1) 1.2 .1 (page 1): Please include the frequency range of these data (i.e., are any data filters applied to the raw data?).

2) 1.2.4 (page 2): Please include the inter-stimulus interval and jitter in the stimulus presentation.

3) 1.2.4 (page 2): Please include the total testing time for each run.

4) 1.2.5 (page 3): Please state that MABC-2 was used to determine motor

abilities and provide the cut-off percentile scores for each group.

5) 1.2.7 (page 3): Please include the test date in the metadata so that the age at testing can be calculated.

6) 1.2.7 (page 3): Any confidential participant information from the metadata (name, email) should be removed. It would be useful to also include the MABC total score or MABC total percentile, level of hearing impairment, and visual correction would be useful to add to the metadata.

7) Figure 1 (page 6): Rather than plotting data with respect to the participant IDs it would be useful to:

a. Plot the individuals by age, since younger children often have more blink artifacts.

b. Color-code the individuals in each group because one would expect the children with DCD to have more eye-blink artifacts.

Level of interest: An article whose findings are important to those with closely related research interests

Quality of written English: Acceptable

Statistical review: No, the manuscript does not need to be seen by a statistician.

Declaration of competing interests:

I declare that I have no competing interests.
