# Supplementary material for: de novo assembly and population genomic survey of natural yeast isolates with the Oxford Nanopore MinION sequencer
Source: Gigascience. 2017 Jan 7;6(2):1–13. doi: 10.1093/gigascience/giw018 (PMC5466710; doi:10.1093/gigascience/giw018)
Supplement: Supplemental material — Additional file 1: Figure S1. Percentage of correct A, T, C, and G homopolymers in Nanopore 2D reads in either R7 reads (a) or R9 reads (b). Additional file 2: Figure S2. Alignment of the Canu assembly. We aligned the most continuous Canu assembly to the reference genome using nucmer and visualized the alignment using the mummer software suite. Additional file 3: Figure S3. Alignment of the Miniasm assembly. We aligned the most continuous Miniasm assembly to the reference genome using nucmer and visualized the alignment using the mummer software suite. Additional file 4: Figure S4. Alignment of the SMARTdenovo assembly. We aligned the most continuous SMARTdenovo assembly to the reference genome using nucmer and visualized the alignment using the mummer software suite. Additional file 5: Figure S5. Alignment of the ABruijn assembly. We aligned the most continuous ABruijn assembly to the reference genome using nucmer and visualized the alignment using the mummer software suite. Additional file 6: Figure S6. Impact of the input coverage used to polish the nanopore-only consensus. Additional file 7: Figure S7. Nanopore 2D reads coverage distribution across all yeast strains. In total 95 MinION MkI runs were done. We obtained a 2D read coverage fluctuating between 25x and 120x. Additional file 8: Figure S8. Reads length distribution of 2D reads across all yeast strains. Additional file 9: Figure S9. Fitness of the 21 yeast isolates in the presence of CuSO4 as a function of the detected number of CUP genes in each strain. Additional file 10: Table S1. Metrics of the reads sets that lead to the best S288C assembly for each software. Additional file 11: Table S2. Metrics of S288C assemblies. Varying coverages of 2D reads and reads corrected by Canu were given as input to Canu, Miniasm, SMARTdenovo, and ABruijn. Only the most contiguous assembly is shown below. Metrics were obtained by aligning the assemblies to the reference genome using Quast. Additional file 12: Table S3. [file giw018_Supp.zip › Supplementary_File1.docx]

***de novo* assembly and population genomic survey of natural yeast isolates with the Oxford Nanopore MinION sequencer**

Benjamin Istace^1^, Anne Friedrich^2^, Léo d’Agata^1^, Sébastien Faye^1^, Emilie Payen^1^, Odette Beluche^1^, Claudia Caradec^2^, Sabrina Davidas^1^, Corinne Cruaud^1^, Gianni Liti^3^, Arnaud Lemainque^1^, Stefan Engelen^1^, Patrick Wincker^1,4,5^, Joseph Schacherer^2,§^, Jean-Marc Aury^1,§^

^1^ Commissariat à l’Energie Atomique et aux Energies Alternatives (CEA), Institut de Génomique (IG), Genoscope, BP5706, 91057 Evry, France

^2^ Department of Genetics, Genomics and Microbiology, University of Strasbourg/CNRS UMR 7156, 67000 Strasbourg, France

^3^ Institute of Research on Cancer and Ageing of Nice (IRCAN), CNRS UMR 7284-INSERM U1081, Faculté de Médecine, Université de Nice Sophia Antipolis, Nice, France.

^4^ Université d’Evry Val d’Essonne, UMR 8030, CP5706, 91057 Evry, France

^5^ Centre National de Recherche Scientifique (CNRS), UMR 8030, CP5706, 91057 Evry, France

^§^Corresponding authors

**Supplementary Figures and Tables**

**Figure S1: Percentage of correct A, T, C and G homopolymers in Nanopore 2D reads in either R7 reads (panel a) or R9 reads (panel b).**


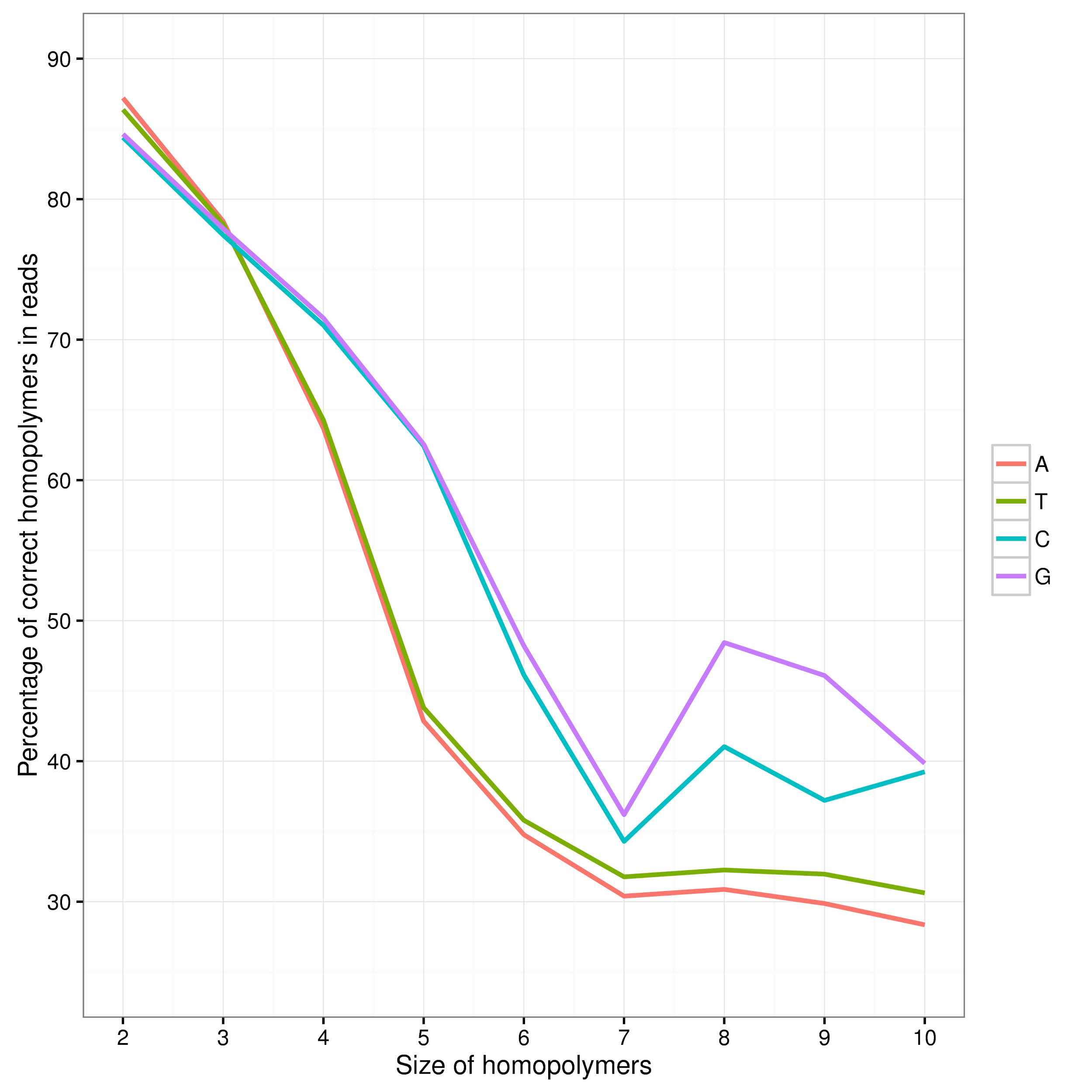

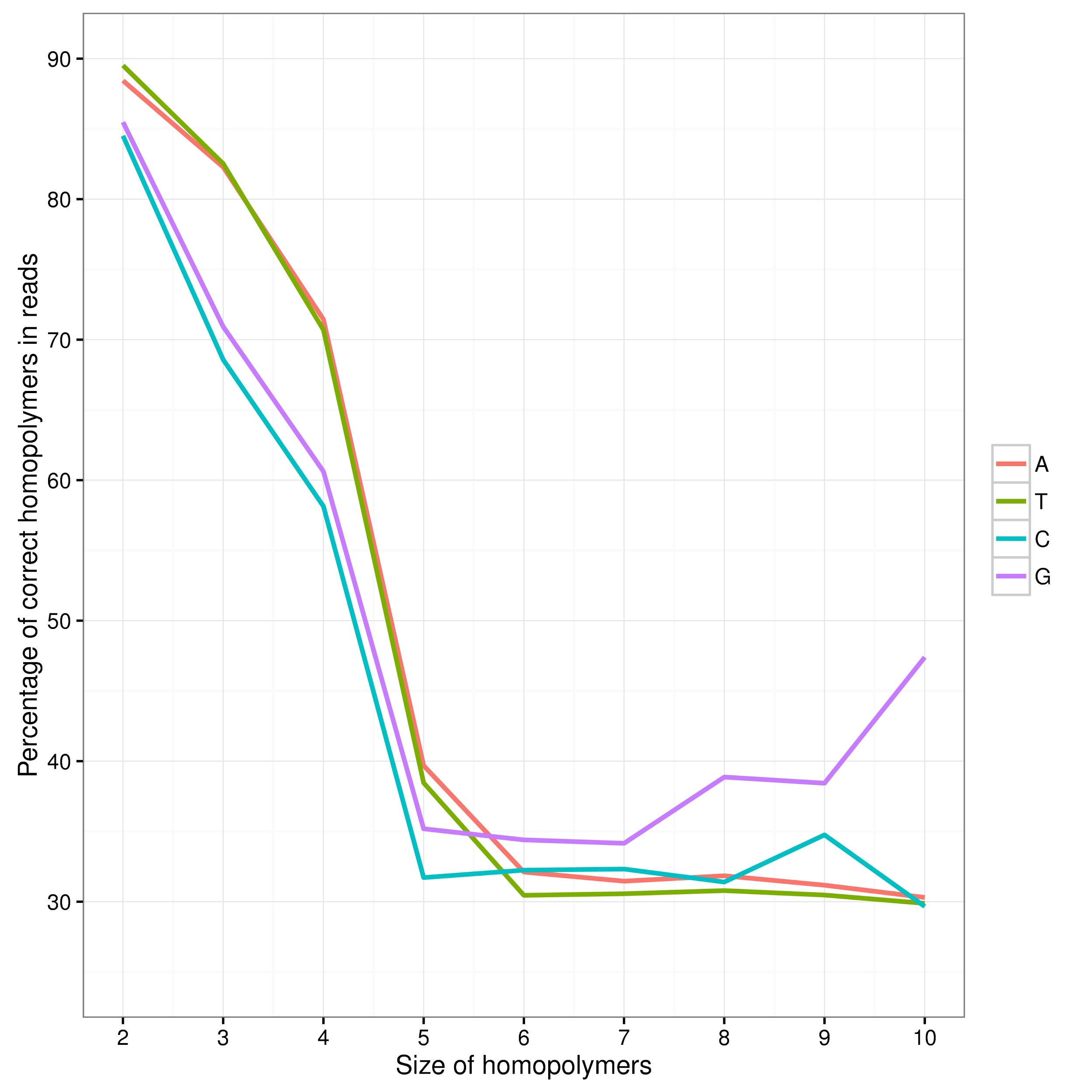


**Figure S2: Alignment of the Canu assembly.** We aligned the most continuous Canu assembly to the reference genome using nucmer and visualized the alignment using the mummer software suite.


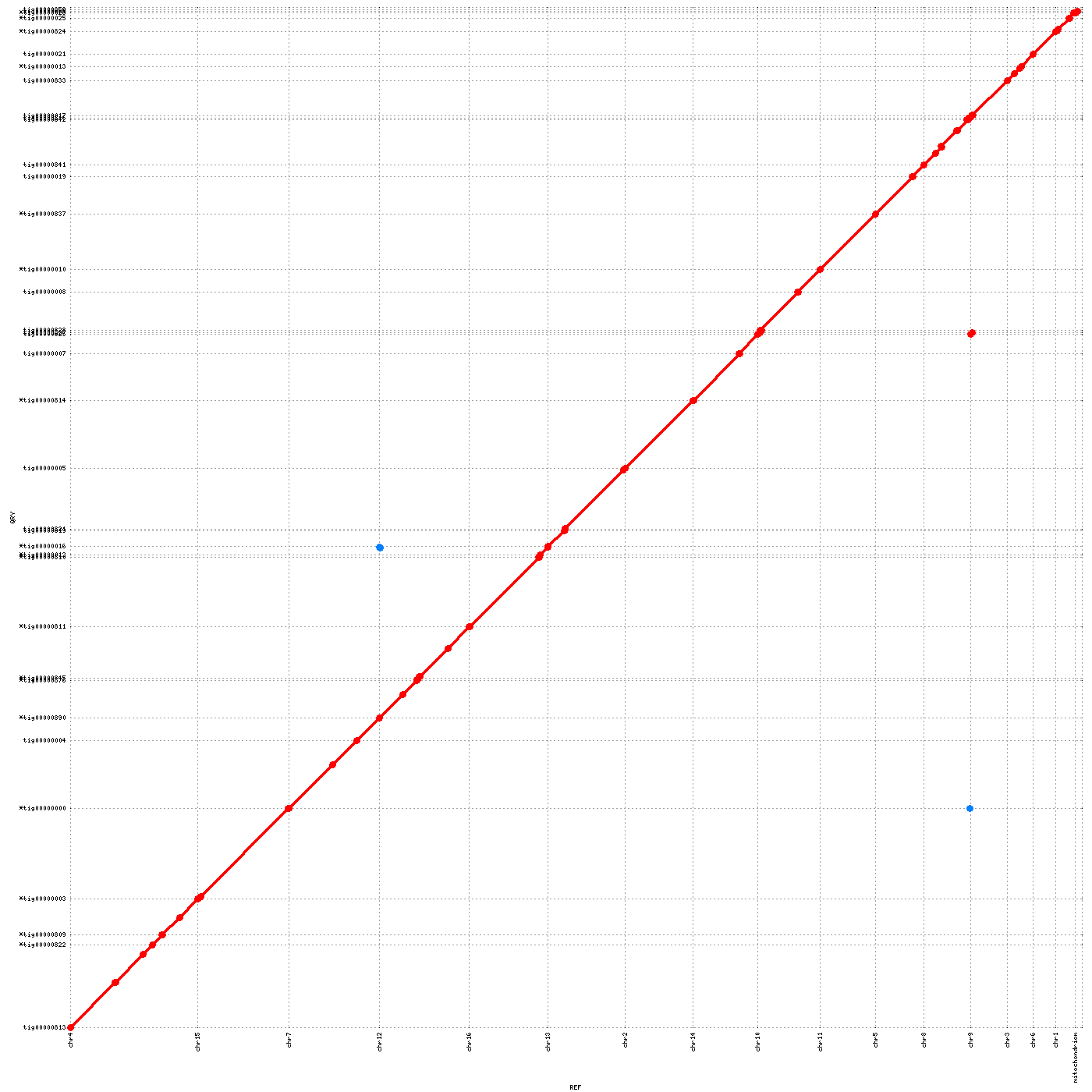


**Figure S3: Alignment of the Miniasm assembly.** We aligned the most continuous Miniasm assembly to the reference genome using nucmer and visualized the alignment using the mummer software suite.


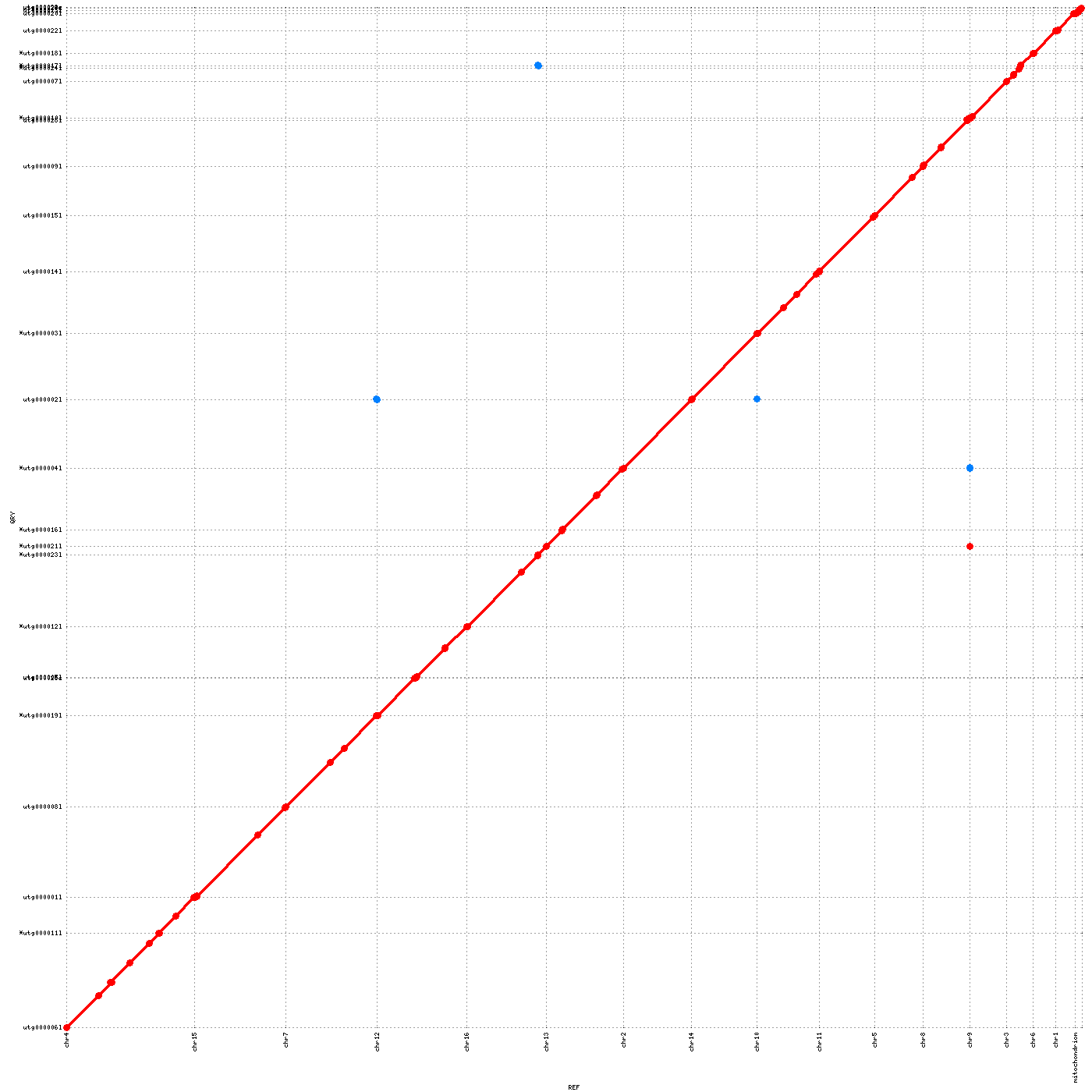


**Figure S4: Alignment of the SMARTdenovo assembly.** We aligned the most continuous SMARTdenovo assembly to the reference genome using nucmer and visualized the alignment using the mummer software suite.


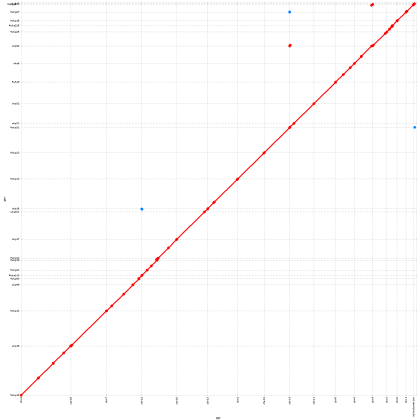


**Figure S5: Alignment of the ABruijn assembly.** We aligned the most continuous ABruijn assembly to the reference genome using nucmer and visualized the alignment using the mummer software suite.


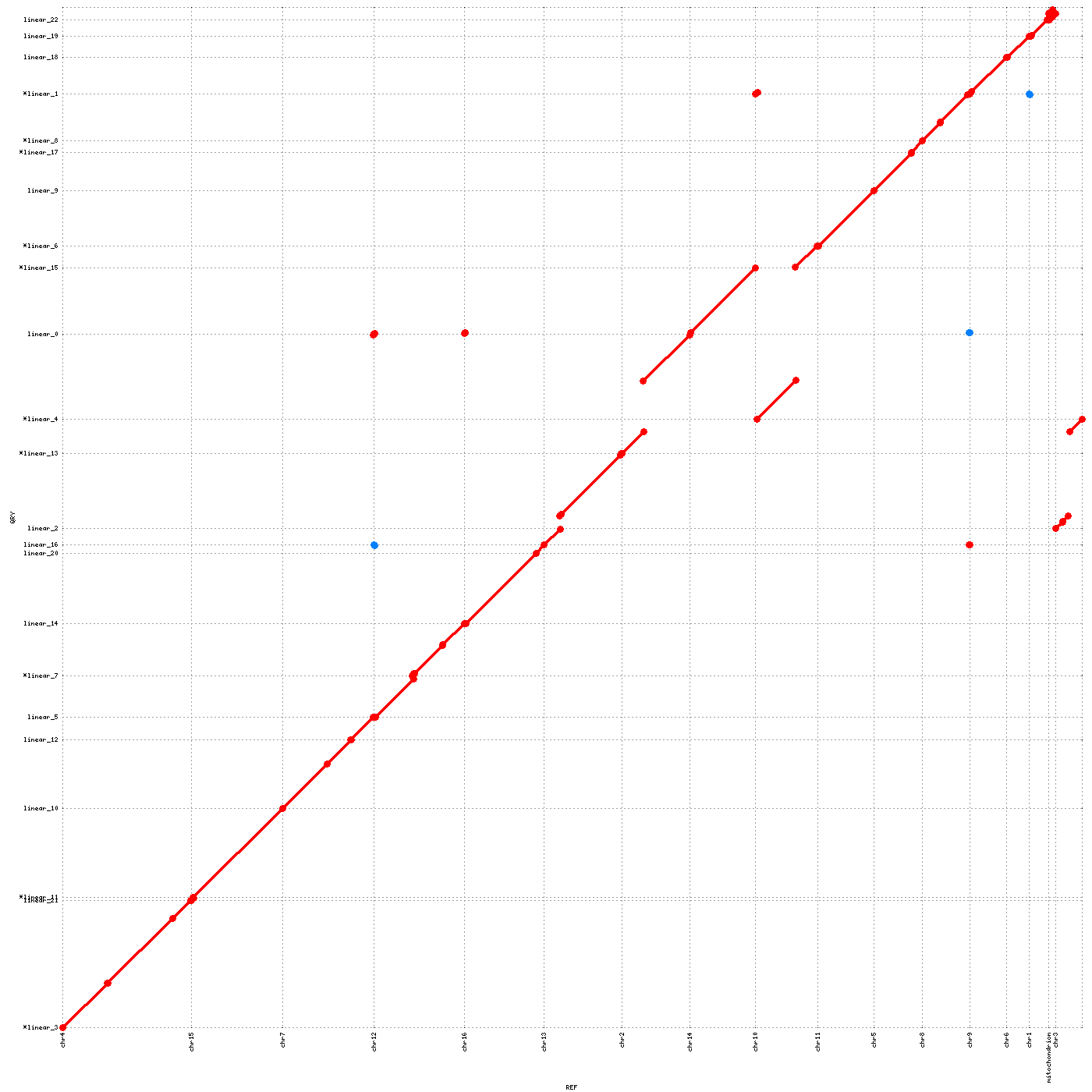


**Figure S6: Impact of the input coverage used to polish the nanopore-only consensus.**

**
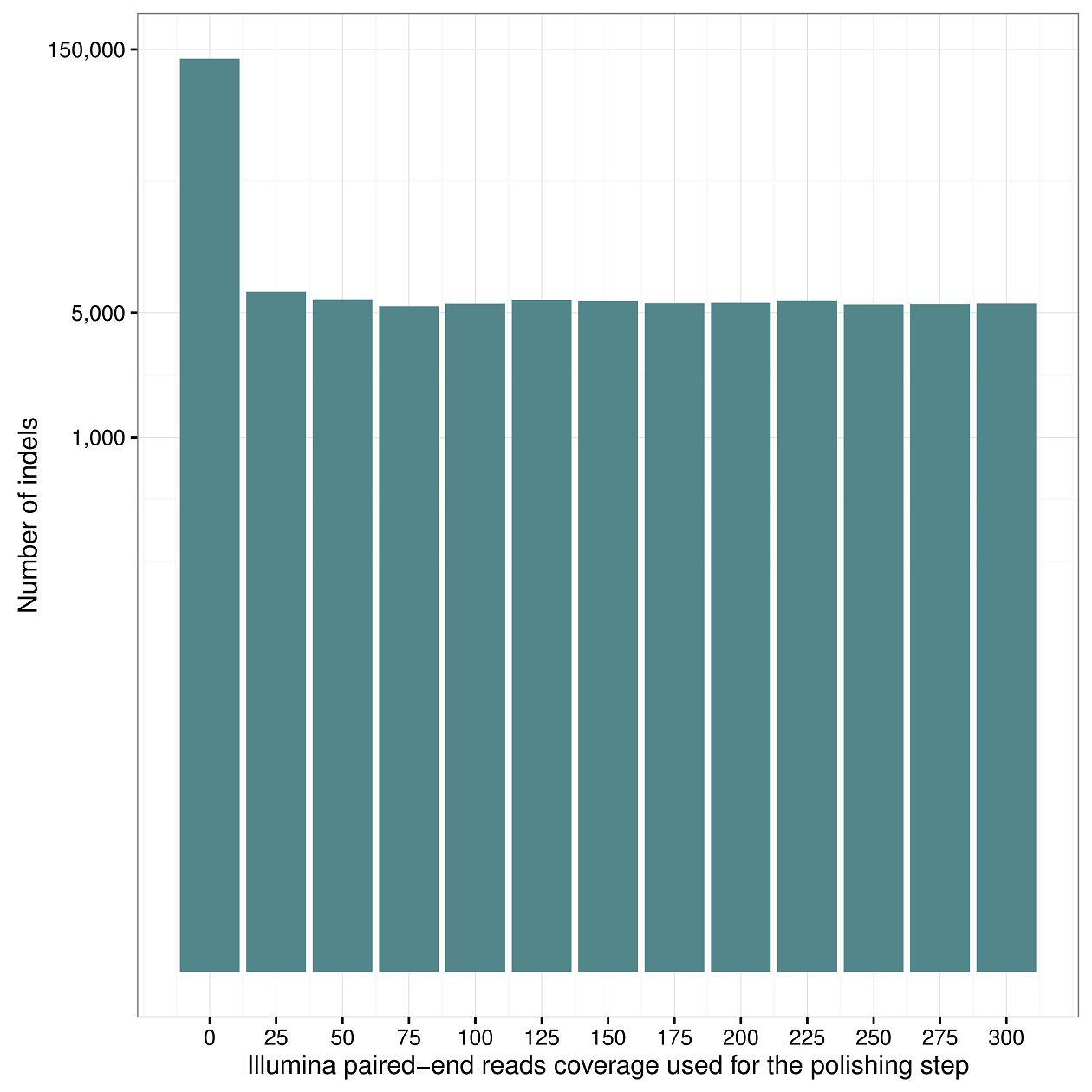
**

**Figure S7: Nanopore 2D reads coverage distribution across all yeast strains.** In total 95 MinION® MkI runs were done. We obtained a 2D reads coverage fluctuating between 25X and 120X.


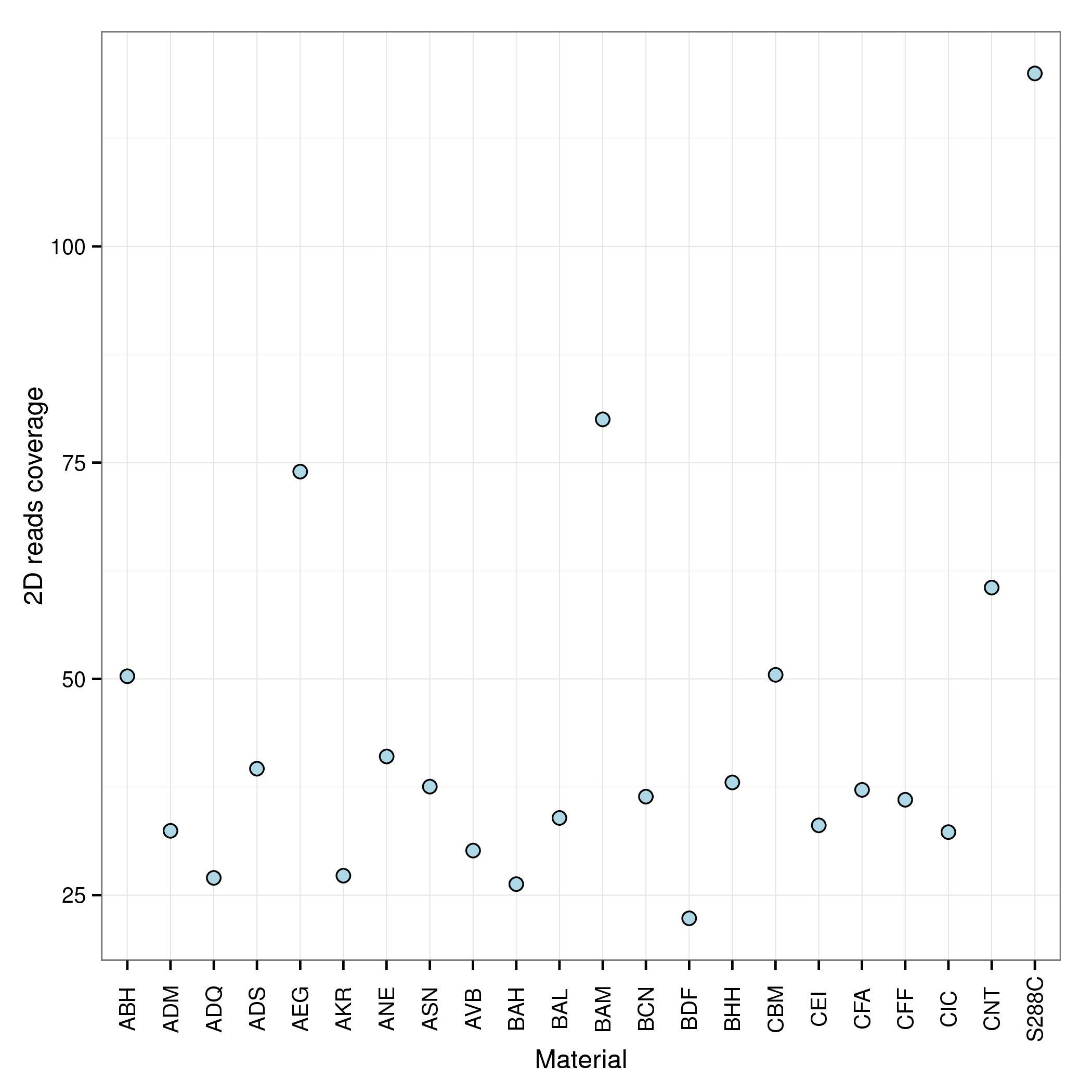


**Figure S8: Reads length distribution of 2D reads across all yeast strains.**


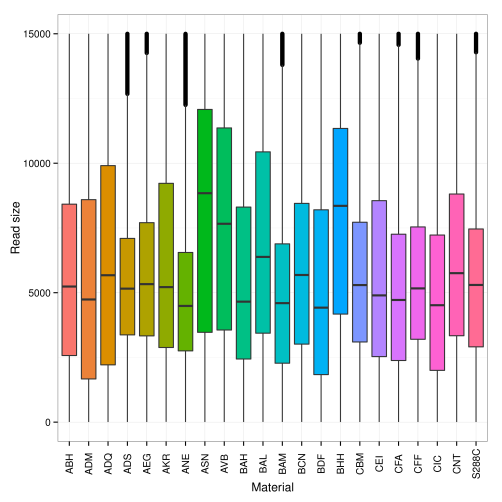


**Figure S9: Fitness of the 21 yeasts isolates in the presence of CuSO_4_** **as a function of the detected number of *CUP* genes in each strain.**


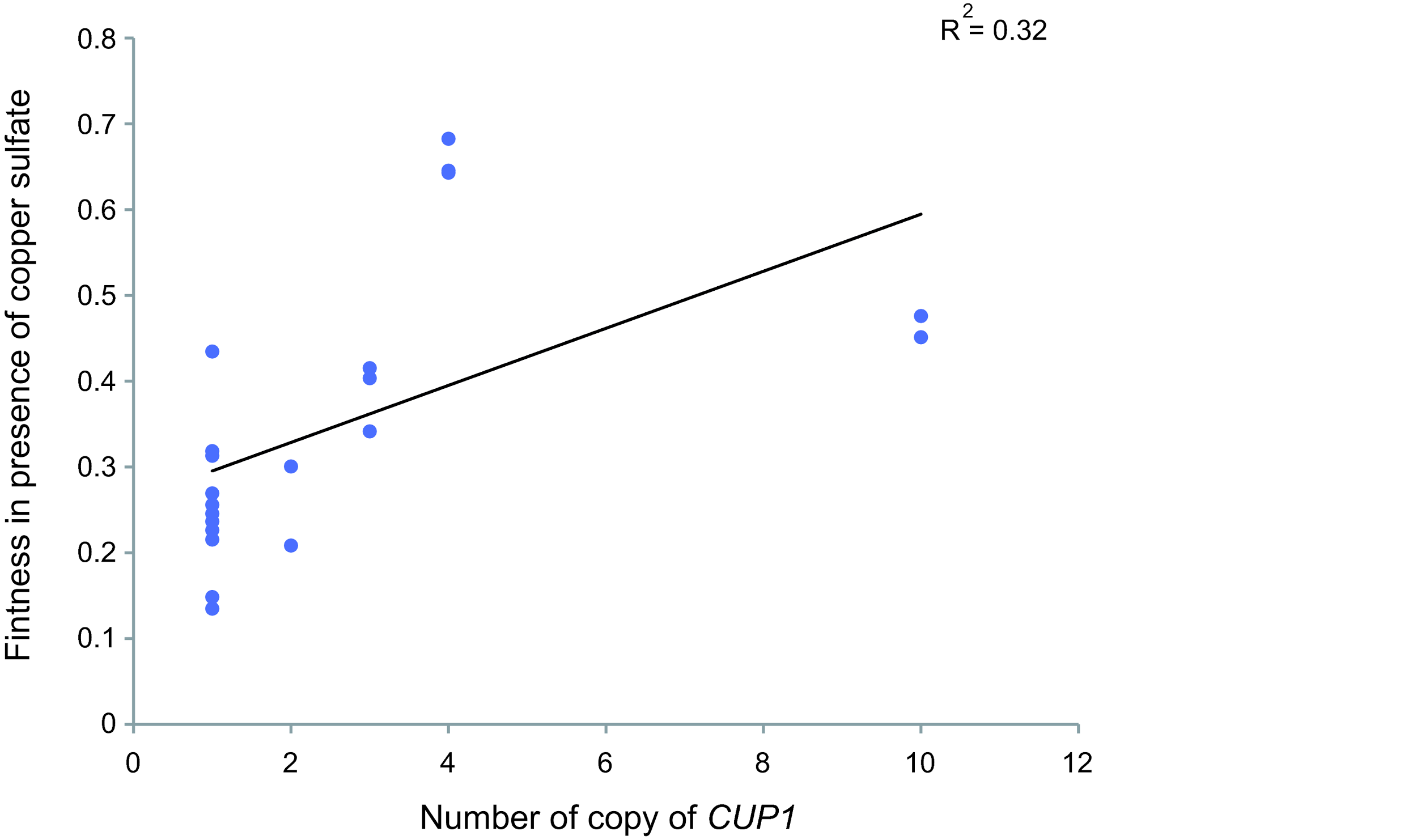


**Table S1:** Metrics of the reads sets that lead to the best S288C assembly for each software.

|  | **Canu** | **Miniasm** | **SMARTdenovo** | **ABruijn** |
| --- | --- | --- | --- | --- |
| Reads set used | 2D pass | Canu-corrected | Longest 2D | 2D |
| Coverage | 67x | 108x | 30x | 120x |
| # reads | 117,773 | 226,274 | 30,225 | 212,539 |
| # reads > 10kb | 16,860 | 21,005 | 28,668 | 28,668 |
| Cumul size (bp) | 822,630,433 | 1,315,936,716 | 369,006,291 | 1,429,441,929 |
| Average size (bp) | 6,984.88 | 5,815.68 | 12,208 | 6,725.55 |
| N50 (bp) | 7,865 | 7,086 | 11,796 | 7,742 |
| Longest read (bp) | 40,954 | 40,076 | 152,333 | 152,333 |

**Table S2: Metrics of S288C assemblies.** Varying coverages of 2D reads and reads corrected by Canu were given as input to Canu, Miniasm, SMARTdenovo and ABruijn. Only the most contiguous assembly is shown below. Metrics were obtained by aligning the assemblies to the reference genome using Quast.

|  | **Canu** | **Miniasm** | **SMARTdenovo** | **ABruijn** |
| --- | --- | --- | --- | --- |
| Reads set used | 2D pass | Canu-corrected | Longest 2D | 2D |
| Coverage | 67x | 108x | 30x | 120x |
| # reads > 10kb | 16,860 | 21,005 | 28,668 | 28,668 |
| # contigs | 37 | 28 | 26 | **23** |
| Cumulative size | 12,037,031 | 11,797,258 | 12,018,244 | 11,981,688 |
| N50 | 601,142 | 717,440 | 771,149 | **803,275** |
| N90 | 188,989 | **258,881** | 238,808 | 253,240 |
| L50 | 8 | 7 | 7 | **6** |
| L90 | 20 | **16** | **16** | **16** |
| # mismatches | **1,259** | 5,718 | 6,970 | 1,557 |
| # insertions | **436** | 2,488 | 5,626 | 584 |
| # deletions | **120,365** | 160,582 | 128,050 | 120,867 |
| # deletions in homopolymers | **70,069** | 107,111 | 79,152 | 71,570 |
| # genes | 6,243 +  19 part | 6,202 +  33 part | **6,251 +**  **24 part** | 6,216 +  26 part |
| # genes without indels | 454 | 514 | 429 | **683** |

**Table S3:** **Number of copy of *CUP1* and *ENA1-2* tandem-repeated genes.** Second column indicates the expected number of copy based on the alignment of Illumina reads on the reference. Other columns indicate either the maximum number found in Nanopore reads or in assemblies.

| **Gene** | **Expected**  **(Illumina coverage)** | **Nanopore Reads** | **Canu** | **Miniasm** | **SMARTdenovo** | **ABruijn** |
| --- | --- | --- | --- | --- | --- | --- |
| *CUP1* | 7 | 8 | 9 | 7 | 7 | 7 |
| *ENA1-2* | 4 | 5 | 3 | 2 | 4 | 4 |

**Table S4: Comparison of SMARTdenovo assemblies using R9 reads.**

| Assembler | SMARTdenovo | | | |
| --- | --- | --- | --- | --- |
| Reads set used | 1D + 2D | 2D | Longest 1D + 2D | Longest 2D |
| Coverage | 83 X | 46 X | 30 X | 30 X |
| Reads > 10 kbp | 16,003 | 7,292 | 16,003 | 7,292 |
| # contigs  (> 1000bp) | 30 | 28 | 23 | 31 |
| Cumul size | 12,208,033 | 12,227,321 | 12,703,721 | 12,332,622 |
| N50 | 679,972 | 675,015 | 789,837 | 582,251 |
| N90 | 218,213 | 225,768 | 446,090 | 191,721 |
| L50 | 8 | 8 | 7 | 8 |
| L90 | 19 | 19 | 15 | 20 |
| # mismatches | 12,999 | 12,394 | 23,024 | 12,527 |
| # indels | 88,976 | 95,012 | 115,059 | 89,388 |
| # genes | 6,257 + 24 part | 6,250 + 22 part | 6,302 + 18 part | 6,290 + 29 part |
| # genes without indels | 1,391 | 848 | 1,226 | 1,374 |

**Table S5: Description of the studied isolates**

| **Alias** | **Isolate name** | **Isolation location** | **Geographical origins** |
| --- | --- | --- | --- |
| ABH | YJM454_1b | Human clinical | NA |
| ADM | CLIB324_2 | Bakery | Vietnam |
| ADQ | K12_2 | Sake | Japan |
| ADS | T73_1b | Monastrel grape fermentation | Spain |
| AEG | CBS7962 | Fermenting concentrated syrup | Brazil |
| AKR | CLQCA_05-006 | Rotten fruit | Ecuador |
| ANE | YPS134 | Soil beneath Quercus velutina | USA |
| ASN | CLIB630 | Dairy cheese | France |
| AVB | CECT10266 | Tanning liquor | Spain |
| BAH | SX3 | Bark from Carya sp. | China |
| BAL | HN6 | Rotten wood | China |
| BAM | HN10 | Rotten wood | China |
| BCN | HE015 | Human feces | French Guiana |
| BDF | #57 | Evolution canyon | Israel |
| BHH | CLIB1071 | Cider brewery | France |
| CBM | 3 | Chalcidoidea | Germany |
| CEI | GE14S01-7B | Soil | Taiwan |
| CFA | Dji2-2A(a) | Palm wine | Djibouti |
| CFF | 6.2_WLP570 | Carlsberg Beer | Belgium |
| CIC | Ponton 11 | Mouth | Spain |
| CNT | RP11.4.11 | Bioethanol | Brazil |
| CRV | S288C | Laboratory strain |  |

**Table S6:** Metrics of the reads sets that lead to the best SMARTdenovo assembly for each strain.

| **Alias** | **Coverage** | **#reads** | **#reads > 10kb** | **Cumul size (bp)** | **Average size (bp)** | **N50 (bp)** | **Longest read (bp)** |
| --- | --- | --- | --- | --- | --- | --- | --- |
| ABH | 40x | 33,543 | 27,695 | 486,280,817 | 14,497 | 14,822 | 119,621 |
| ADM | 34x | 56,797 | 16,291 | 418,477,277 | 7,367 | 10,610 | 58,651 |
| ADQ | 27x | 34,290 | 15,539 | 328,206,508 | 9,571 | 12,324 | 45,283 |
| ADS | 30x | 41,219 | 9,117 | 364,715,802 | 8,848 | 8,650 | 50,262 |
| AEG | 50x | 59,950 | 21,931 | 607,853,420 | 10,139 | 10,147 | 131,247 |
| AKR | 28x | 39,984 | 13,375 | 331,237,393 | 8,284 | 11,730 | 49,331 |
| ANE | 41x | 90,188 | 6,549 | 499,085,175 | 5,533 | 6,356 | 269,577 |
| ASN | 37x | 32,377 | 25,002 | 456,523,377 | 14,100 | 15,761 | 75,806 |
| AVB | 30x | 34,236 | 18,859 | 366,582,387 | 10,707 | 13,452 | 53,690 |
| BAH | 26x | 38,430 | 12,092 | 319,452,878 | 8,312 | 11,826 | 62,951 |
| BAL | 34x | 44,747 | 18,777 | 412,409,008 | 9,216 | 12,241 | 51,867 |
| BAM | 40x | 45,882 | 18,519 | 486,285,428 | 10,598 | 10,173 | 77,149 |
| BCN | 36x | 53,308 | 14,000 | 442,420,733 | 8,299 | 10,174 | 53,577 |
| BDF | 22x | 25,258 | 11,075 | 271,371,158 | 10,744 | 15,163 | 78,821 |
| BHH | 38x | 40,901 | 24,214 | 462,160,252 | 11,299 | 12,921 | 45,738 |
| CBM | 40x | 53,066 | 14,808 | 486,284,216 | 9,163 | 9,228 | 43,072 |
| CEI | 33x | 37,070 | 15,873 | 401,993,423 | 10,844 | 16,888 | 58,862 |
| CFA | 37x | 67,278 | 11,564 | 451,897,031 | 6,716 | 8,088 | 34,654 |
| CFF | 36x | 66,417 | 10,199 | 438,020,761 | 6,595 | 7,915 | 84,147 |
| CIC | 33x | 66,826 | 9,165 | 403,318,855 | 6,035 | 7,784 | 57,896 |
| CNT | 50x | 56,103 | 26,001 | 607,853,543 | 10,834 | 11,604 | 49,167 |
| S288C | 30x | 30,225 | 28,668 | 369,006,291 | 12,208 | 11,796 | 152,333 |
