# Supplementary material for: de novo assembly and population genomic survey of natural yeast isolates with the Oxford Nanopore MinION sequencer
Source: Gigascience. 2017 Jan 7;6(2):1–13. doi: 10.1093/gigascience/giw018 (PMC5466710; doi:10.1093/gigascience/giw018)
Supplement: Response_to_Reviewer_Comments_Original_Submission.pdf [file giw018_Response_to_Reviewer_Comments_Original_Submission.pdf]

Reviewer's report
Title: de novo assembly and population genomic survey of natural yeast isolates with the Oxford Nanopore MinION sequencer

Reviewer number: 1
Reviewer's report:
The authors describe assembly experiments on a set of yeast isolates using Oxford Nanopore MinION technology, both the older (and now discontinued) R7.3 and the newer (but about to be superceded) R9 chemistries. The methods are well-described, the data has been deposited in a stable archive and the results section performs a number of useful assessments of the quality of the assemblies using different de novo assembly tools.

Table S2 appears to be a subset of the columns of Table 1. If it doesn't provide any additional information, it shoudl be dropped.

Answer: Table S2 contains the metrics of the raw nanopore assemblies while Table 1 contains the metrics of the post-polished assemblies. We kept Table S2 in the supplementary data.

I would prefer that Table S4 be moved out of the supplement and into the main article. Details on polishing effects are important for understanding the ONT platform, and therefore it is unfortunate to bury them in the supplement. I might also argue that Tables S8 and S9 are unfortunate to maroon in the supplement, as these are demonstrating the value of the long read assembly.

Answer: We moved Tables S4, S8 and S9 in the main text.

Reviewer number: 2
Reviewer's report:
Authors present a survey on de novo assembly of yeast genomes using Oxford Nanopore MinION sequencer. Authors assembled a total of 22 yeast strains, the Saccharomyces cerevisiae S288C used to asses the quality and performances of the assemblers and data, and 21 strains selected for their diversity and spread. They compare various types of data that can be produced with MiniION (e.g. 2D and 1D reads) and different MIniIon chemestries (R7.3 and the more recent R9).

Data is assembled using 4 different MiniIon only assemblers: Canu], Miniasm, SMARTdenovo and ABruijn.

They perform many assemblies with different types of data as input. They also use Illumina read to error correct the final assembly with Pilon.

In general I like the paper, it is a snapshot of the current status of de novo assembly with MiniIon and gives the possibility to a reader to have an idea of what tools to use and what results to expect.

I have some concerns that I want the authors to address:
- they often say in the text "kept the best assembly for each software" (e.g., page 6 line 141). They employ many metrics to discuss about assembly (contiguity, gene coverage, indels) and I like it a lot, but it is not clear how they select the "best" assembly. If for example they choose the best assembly based only on contiguity they might be constantly choosing assemblies affected by many errors, while less contiguous assemblies might be characterized by more correct sequences

Answer: We modified the text at several locations (lines 143,144,150,155,159 and 228) and we replaced “best assembly” by “most contiguous assembly”. Indeed, we selected the best assembly for each method

based on contiguity metrics (N50, Number of contigs and cumulative size).

- page 6 line 152: "a high proportion of mismateches and indels" : this needs to be more specific, what is "high proportion"?

Answer: We modified the corresponding sentence (lines 153-154), and replaced “a high proportion of mismatches and indels” by “the higher number of mismatches and indels”. Furthermore we added a reference to Table S2 which contains the metrics (number of mismatches and indels) of the nanopore-only assemblies before the polishing step.

- The abstract is pretty positive about using only MiniIon data in de novo assembly, or at least that is my impression. Moreover, from the abstract and from the introduction part I was expecting to read about a MiniIon only evaluation and comparison. Instead, the assemblies presented in Table 1 and the various discussions on the evaluation show that all MiniIon assemblies needed Pilon (and therefore the 300X Illumina coverage) to be corrected. Moreover, to finish up the gneomes 8Kbp and 20Kbp library have been used, and I assume these are MP Illumina libraries. Therefore, I am now pretty skeptical about the ability of miniion to assemble alone yeast genome... I think that the abstract needs to be toned down and pint point more the need of complementary technologies to obtain a final assembly.

Answer: We take into account the reviewer comments and change the last sentence of the abstract (lines 56-59) to better reflect current issues with nanopore-only assemblies. All the assemblies were based on nanopore and Illumina paired-end sequencing; we didn’t sequence any mate-pairs data. The nanopore-only assemblies show an accurate structure (organization of genomic elements, like genes or transposons) but the final consensus of those assemblies still remains problematic.

I want to point out this paper (I am one of the authors)
https://gigascience.biomedcentral.com/articles/10.1186/s13742-015-0094-1
In this paper a multi-technology approch is followed combining Illumina, PacBio, and Optical Maps on an yeast genome. In case you have a similar variaty to the one assembled in this paper would be nice to compare the assembly presented in the paper with the MiniIon assembly... This is a plus, but I think it would really show potentials of MiniIon.

Answer: It would be very interesting to compare the results of a MinION sequencing strategy and the multi-technology approach (i.e. Illumina, PacBio and Optical Map), which was used to assemble the genome of the Dekkera bruxellensis yeast in Olsen et al. - GigaScience 2015. However, because we sequenced Saccharomyces cerevisiae genomes in our study, this is something impossible. Indeed, Dekkera bruxellensis and Saccharomyces cerevisiae are not closely related species and their genomes are very different in terms of chromosome number and there is no conservation of synteny at all.

Reviewer number: 3
Reviewer's report:
This is an excellent, timely, and well put together study. The results will be greatly helpful to many working on integrating this technology into the genome sequencing ecosystem.

Lines 195-196 describe read polishing with Pilon. It would be helpful to indicate what this depth of coverage was used to polish with the 2x250bp - I realize its in the table legend but could be helpful to include in the text here. also might be helpful to know if 300x is really needed to correct / polish well - would 100x work equally well?

Answer: We modified the text accordingly; we added the Illumina coverage that was used during the polishing step (line 198). Concerning the optimal coverage, we agreed with the reviewer and we performed several polishing with subsets of Illumina PE reads (from 25X to 300X). We added several sentences in the

text (lines 205-209) and a supplementary figure (Figure S6) to show that a low coverage (100X or less) is sufficient to correct the consensus of nanopore-only assemblies.

Lines 209-216. Comparing the SPAdes Illumina assembly to the Nanopore only assembly -- The Table presents the QUAST(?) results that gene completeness is actually lower in the Illumina-only assembly but these are mostly indel free? Could be mentioned in the text here?

Answer: We added a sentence to underline these features (lines 214-216).

Doesn't SPAdes also have a option for co-assembly with Illumina + MinION data? Did this produce a useful / comparable assembly ?

Answer: That's right, but we'd like to focus our comparison on nanopore-only assemblers. However, we launched Spades with Illumina and nanopore data. The output assembly was composed of 143 contigs with a N50 near 250Kb. Although the assembly is less fragmented than the Illumina-only assembly it still remains highly fragmented compared to nanopore-only assemblies.

Lines 240 - 257. Sequencing the additional strains. It was unclear how the Pilon polishing is done here - the authors say 300x Illumina paired-end reads - are these from the same strain? Were illumina libraries made and sequenced for each strain or was this using the 1002 genome data ? (the 1002 site says it used 2x102 bp?)

Answer: We modified the text to better describe the pilon process (line 257) and we added a section in the method chapter to explain how the 22 genomes have been assembled (lines 504-509).

One idea I had in reading the manuscript. An additional type of repeat variation that is seen in Saccharomyces and other yeasts is the changes regarding simple repeats. These are particularly interesting in context when they fall within context of genic region generating instability that leads to phenotypic variation as the authors I am sure are aware. This was explored through PCR and sequencing in multiple strains by Verstrepen et al Nat Gen 2005 - in particular FLO1 has variable repeated regions easy to pick out. I searched FLO1 against the assemblies and found nice example of expanded repeat in the gene either matching the FLO5 or FLO1 copies. I worked up the example here
https://gist.github.com/hyphaltip/9f5256854f7a049ad81847c4740ece94#file-flo_loci-table
So it looks like there is variability in the size of the repeats in a few of these strains. Up to the authors if this is worth remarking on but it might be something that could also be better resolved than in Illumina assembly.

Answer: This is definitively an interesting comment. Indeed, there are repeated regions in the FLO1 gene, which additionally have an impact on the phenotypic diversity (e.g. adhesion, flocculation or biofilm formation). FLO1 is 4.6 kb long and contains a variable number of repeats of approx. 100 nt, separated by a 45-nt sequence. Consequently, these repeated structures having a small size can be resolved using Illumina sequencing data. In the frame of our study, we really wanted to focus on larger repeated structures, i.e. involving entire genes such as ENA and CUP genes tandem arrays. Indeed, long read sequencing technologies should have a high resolution compared to short read strategies.

Excellent description of methods, versions of software used, and providing reproducible methods. Though it changes rarely, it may be useful to spell out the exact version of the S288C genome assembly and GFF files used in validation.

Answer: We'd like to thank the reviewer for its conscientious reading of the manuscript as well as its suggestions of improvements.
