# Supplementary material for: de novo assembly and population genomic survey of natural yeast isolates with the Oxford Nanopore MinION sequencer
Source: Gigascience. 2017 Jan 7;6(2):1–13. doi: 10.1093/gigascience/giw018 (PMC5466710; doi:10.1093/gigascience/giw018)
Supplement: Response_to_Reviewer_Comments_Revision_1.pdf [file giw018_Response_to_Reviewer_Comments_Revision_1.pdf]

Edits made addressing final comments and DOI integrated.
