# Supplementary material for: de novo assembly and population genomic survey of natural yeast isolates with the Oxford Nanopore MinION sequencer
Source: Gigascience. 2017 Jan 7;6(2):1–13. doi: 10.1093/gigascience/giw018 (PMC5466710; doi:10.1093/gigascience/giw018)
Supplement: Reviewer_1_Report_Original_Submission.pdf [file giw018_Reviewer_1_Report_Original_Submission.pdf]

Reviewer Report

Title: "de novo assembly and population genomic survey of natural yeast isolates with the Oxford Nanopore MinION sequencer"

Version: Original Submission **Date:** 10/4/2016

Reviewer name: Keith Robison

Reviewer Comments to Author:

The authors describe assembly experiments on a set of yeast isolates using Oxford Nanopore MinION technology, both the older (and now discontinued) R7.3 and the newer (but about to be superceded) R9 chemistries. The methods are well-described, the data has been deposited in a stable archive and the results section performs a number of useful assessments of the quality of the assemblies using different de novo assembly tools.

Table S2 appears to be a subset of the columns of Table 1. If it doesn't provide any additional information, it shoudl be dropped.

I would prefer that Table S4 be moved out of the supplement and into the main article. Details on polishing effects are important for understanding the ONT platform, and therefore it is unfortunate to bury them in the supplement. I might also argue that Tables S8 and S9 are unfortunate to maroon in the supplement, as these are demonstrating the value of the long read assembly.

Methods

Are the methods appropriate to the aims of the study, are they well described, and are necessary controls included? Yes

Conclusions

Are the conclusions adequately supported by the data shown? Yes

Reporting Standards

Does the manuscript adhere to the journal's guidelines on minimum standards of reporting? Yes

Statistics

Are you able to assess all statistics in the manuscript, including the appropriateness of statistical tests used? There are no statistics in the manuscript.

Quality of Written English

Please indicate the quality of language in the manuscript: Acceptable

Declaration of Competing Interests

Please complete a declaration of competing interests, considering the following questions:

- Have you in the past five years received reimbursements, fees, funding, or salary from an organisation that may in any way gain or lose financially from the publication of this manuscript, either now or in the future?
- Do you hold any stocks or shares in an organisation that may in any way gain or lose financially from the publication of this manuscript, either now or in the future?
- Do you hold or are you currently applying for any patents relating to the content of the manuscript?
- Have you received reimbursements, fees, funding, or salary from an organization that holds or has applied for patents relating to the content of the manuscript?
- Do you have any other financial competing interests?
- Do you have any non-financial competing interests in relation to this paper?

If you can answer no to all of the above, write 'I declare that I have no competing interests' below. If your reply is yes to any, please give details below.

I am, like the authors, a participant in the ONT MAP. That's meant free reagents, but also time wasted on bad free reagents, so it's really a wash.

I agree to the open peer review policy of the journal. I understand that my name will be included on my report to the authors and, if the manuscript is accepted for publication, my named report including any attachments I upload will be posted on the website along with the authors' responses. I agree for my report to be made available under an Open Access Creative Commons CC-BY license (http://creativecommons.org/licenses/by/4.0/). I understand that any comments which I do not wish to be included in my named report can be included as confidential comments to the editors, which will not be published.

I agree to the open peer review policy of the journal

To further support our reviewers, we have joined with Publons, where you can gain additional credit to further highlight your hard work (see: https://publons.com/journal/530/gigascience). On publication of this paper, your review will be automatically added to Publons, you can then choose whether or not to claim your Publons credit. I understand this statement.

Yes
