# Supplementary material for: de novo assembly and population genomic survey of natural yeast isolates with the Oxford Nanopore MinION sequencer
Source: Gigascience. 2017 Jan 7;6(2):1–13. doi: 10.1093/gigascience/giw018 (PMC5466710; doi:10.1093/gigascience/giw018)
Supplement: Reviewer_2_Report_Original_Submission.pdf [file giw018_Reviewer_2_Report_Original_Submission.pdf]

Reviewer Report

Title: "de novo assembly and population genomic survey of natural yeast isolates with the Oxford Nanopore MinION sequencer"

Version: Original Submission **Date:** 10/19/2015

Reviewer name: Francesco Vezzi

Reviewer Comments to Author:

Authors present a survey on de novo assembly of yeast genomes using Oxford Nanopore MinION sequencer.

Authors assembled a total of 22 yeast strains, the Saccharomyces cerevisiae S288C used to asses the quality and performances of the assemblers and data, and 21 strains selected for their diversity and spread.

They compare various types of data that can be produced with MiniION (e.g. 2D and 1D reads) and different MIniIon chemestries (R7.3 and the more recent R9).

Data is assembled using 4 different MiniIon only assemblers: Canu], Miniasm, SMARTdenovo and ABruijn

They perform many assemblies with different types of data as input. They also use Illumina read to error correct the final assembly with Pilon.

In general I like the paper, it is a snapshot of the current status of de novo assembly with MiniIon and gives the possibility to a reader to have an idea of what tools to use and what results to expect.

I have some concerns that I want the authors to address:

- they often say in the text "kept the best assembly for each software" (e.g., page 6 line 141). They employ many metrics to discuss about assembly (contiguity, gene coverage, indels) and I like it a lot, but it is not clear how they select the "best" assembly. If for example they choose the best assembly based only on contiguity they might be constantly choosing assemblies affected by many errors, while less contiguous assemblies might be characterized by more correct sequences

- page 6 line 152: "a high proportion of mismateches and indels" : this needs to be more specific, what is "high proportion"?

- The abstract is pretty positive about using only MiniIon data in de novo assembly, or at least that is my impression. Moreover, from the abstract and from the introduction part I was expecting to read about a MiniIon only evaluation and comparison. Instead, the assemblies presented in Table 1 and the various discussions on the evaluation show that all MiniIon assemblies needed Pilon (and therefore the 300X Illumina coverage) to be corrected. Moreover, to finish up the gneomes 8Kbp and 20Kbp library have been used, and I assume these are MP Illumina libraries. Therefore, I am now pretty skeptical about the ability of miniion to assemble alone yeast genome... I think that the abstract needs to be toned down and pint point more the need of complementary technologies to obtain a final assembly.

I want to point out this paper (I am one of the authors)
https://gigascience.biomedcentral.com/articles/10.1186/s13742-015-0094-1

In this paper a multi-technology approch is followed combining Illumina, PacBio, and Optical Maps on an yeast genome. In case you have a similar variaty to the one assembled in this paper would be nice to compare the assembly presented in the paper with the MiniIon assembly... This is a plus, but I think it would really show potentials of MiniIon.

Methods

Are the methods appropriate to the aims of the study, are they well described, and are necessary controls included? Yes

Conclusions

Are the conclusions adequately supported by the data shown? Yes

Reporting Standards

Does the manuscript adhere to the journal's guidelines on minimum standards of reporting? Yes

Statistics

Are you able to assess all statistics in the manuscript, including the appropriateness of statistical tests used? There are no statistics in the manuscript.

Quality of Written English

Please indicate the quality of language in the manuscript: Acceptable

Declaration of Competing Interests

Please complete a declaration of competing interests, considering the following questions:

- Have you in the past five years received reimbursements, fees, funding, or salary from an organisation that may in any way gain or lose financially from the publication of this manuscript, either now or in the future?
- Do you hold any stocks or shares in an organisation that may in any way gain or lose financially from the publication of this manuscript, either now or in the future?
- Do you hold or are you currently applying for any patents relating to the content of the manuscript?
- Have you received reimbursements, fees, funding, or salary from an organization that holds or has applied for patents relating to the content of the manuscript?
- Do you have any other financial competing interests?
- Do you have any non-financial competing interests in relation to this paper?

If you can answer no to all of the above, write 'I declare that I have no competing interests' below. If your reply is yes to any, please give details below.

I declare that I have no competing interests

I agree to the open peer review policy of the journal. I understand that my name will be included on my report to the authors and, if the manuscript is accepted for publication, my named report including any attachments I upload will be posted on the website along with the authors' responses. I agree for my report to be made available under an Open Access Creative Commons CC-BY license (http://creativecommons.org/licenses/by/4.0/). I understand that any comments which I do not wish to be included in my named report can be included as confidential comments to the editors, which will not be published.

I agree to the open peer review policy of the journal

To further support our reviewers, we have joined with Publons, where you can gain additional credit to further highlight your hard work (see: https://publons.com/journal/530/gigascience). On publication of this paper, your review will be automatically added to Publons, you can then choose whether or not to claim your Publons credit. I understand this statement.

Yes
