# Supplementary material for: de novo assembly and population genomic survey of natural yeast isolates with the Oxford Nanopore MinION sequencer
Source: Gigascience. 2017 Jan 7;6(2):1–13. doi: 10.1093/gigascience/giw018 (PMC5466710; doi:10.1093/gigascience/giw018)
Supplement: Reviewer_3_Report_Original_Submission.pdf [file giw018_Reviewer_3_Report_Original_Submission.pdf]

**Reviewer Report**

**Title:** "de novo assembly and population genomic survey of natural yeast isolates with the Oxford Nanopore MinION sequencer"

**Version:** Original Submission **Date:** 9/26/2016

**Reviewer name:** Jason Stajich

**Reviewer Comments to Author:**

This is an excellent, timely, and well put together study. The results will be greatly helpful to many working on integrating this technology into the genome sequencing ecosystem.

Lines 195-196 describe read polishing with Pilon. It would be helpful to indicate what this depth of coverage was used to polish with the 2x250bp - I realize its in the table legend but could be helpful to include in the text here. also might be helpful to know if 300x is really needed to correct / polish well - would 100x work equally well?

Lines 209-216. Comparing the SPAdes Illumina assembly to the Nanopore only assembly -- The Table presents the QUAST(?) results that gene completeness is actually lower in the Illumina-only assembly but these are mostly indel free? Could be mentioned in the text here?

Doesn't SPAdes also have a option for co-assembly with Illumina + MinION data? Did this produce a useful / comparable assembly ?

Lines 240 - 257. Sequencing the additional strains. It was unclear how the Pilon polishing is done here - the authors say 300x Illumina paired-end reads - are these from the same strain? Were illumina libraries made and sequenced for each strain or was this using the 1002 genome data ? (the 1002 site says it used 2x102 bp?)

One idea I had in reading the manuscript. An additional type of repeat variation that is seen in Saccharomyces and other yeasts is the changes regarding simple repeats. These are particularly interesting in context when they fall within context of genic region generating instability that leads to phenotypic variation as the authors I am sure are aware. This was explored through PCR and sequencing in multiple strains by Verstrepen et al Nat Gen 2005 - in particular FLO1 has variable repeated regions

easy to pick out. I searched FLO1 against the assemblies and found nice example of expanded repeat in the gene either matching the FLO5 or FLO1 copies. I worked up the example here

https://gist.github.com/hyphaltip/9f5256854f7a049ad81847c4740ece94#file-flo_loci-table

So it looks like there is variability in the size of the repeats in a few of these strains. Up to the authors if this is worth remarking on but it might be something that could also be better resolved than in Illumina assembly.

Excellent description of methods, versions of software used, and providing reproducible methods. Though it changes rarely, it may be useful to spell out the exact version of the S288C genome assembly and GFF files used in validation.

**Methods**

Are the methods appropriate to the aims of the study, are they well described, and are necessary controls included? Yes

**Conclusions**

Are the conclusions adequately supported by the data shown? Yes

**Reporting Standards**

Does the manuscript adhere to the journal's guidelines on minimum standards of reporting? Yes

**Statistics**

Are you able to assess all statistics in the manuscript, including the appropriateness of statistical tests used? There are no statistics in the manuscript.

**Quality of Written English**

Please indicate the quality of language in the manuscript: Acceptable

**Declaration of Competing Interests**

Please complete a declaration of competing interests, considering the following questions:

- Have you in the past five years received reimbursements, fees, funding, or salary from an organisation that may in any way gain or lose financially from the publication of this manuscript, either now or in the future?
- Do you hold any stocks or shares in an organisation that may in any way gain or lose financially from the publication of this manuscript, either now or in the future?

- Do you hold or are you currently applying for any patents relating to the content of the manuscript?
- Have you received reimbursements, fees, funding, or salary from an organization that holds or has applied for patents relating to the content of the manuscript?
- Do you have any other financial competing interests?
- Do you have any non-financial competing interests in relation to this paper?

If you can answer no to all of the above, write 'I declare that I have no competing interests' below. If your reply is yes to any, please give details below.

I declare that I have no competing interests

I agree to the open peer review policy of the journal. I understand that my name will be included on my report to the authors and, if the manuscript is accepted for publication, my named report including any attachments I upload will be posted on the website along with the authors' responses. I agree for my report to be made available under an Open Access Creative Commons CC-BY license (http://creativecommons.org/licenses/by/4.0/). I understand that any comments which I do not wish to be included in my named report can be included as confidential comments to the editors, which will not be published.

I agree to the open peer review policy of the journal

To further support our reviewers, we have joined with Publons, where you can gain additional credit to further highlight your hard work (see: https://publons.com/journal/530/gigascience). On publication of this paper, your review will be automatically added to Publons, you can then choose whether or not to claim your Publons credit. I understand this statement.

Yes
